# The clinical and genetic spectrum of inherited glycosylphosphatidylinositol deficiency disorders

**DOI:** 10.1093/brain/awae056

**Published:** 2024-03-08

**Authors:** Jai Sidpra, Sniya Sudhakar, Asthik Biswas, Flavia Massey, Valentina Turchetti, Tracy Lau, Edward Cook, Javeria Raza Alvi, Hasnaa M Elbendary, Jerry L Jewell, Antonella Riva, Alessandro Orsini, Aglaia Vignoli, Zara Federico, Jessica Rosenblum, An-Sofie Schoonjans, Matthias de Wachter, Ignacio Delgado Alvarez, Ana Felipe-Rucián, Nourelhoda A Haridy, Shahzad Haider, Mashaya Zaman, Selina Banu, Najwa Anwaar, Fatima Rahman, Shazia Maqbool, Rashmi Yadav, Vincenzo Salpietro, Reza Maroofian, Rajan Patel, Rupa Radhakrishnan, Sanjay P Prabhu, Klaske Lichtenbelt, Helen Stewart, Yoshiko Murakami, Ulrike Löbel, Felice D’Arco, Emma Wakeling, Wendy Jones, Eleanor Hay, Sanjay Bhate, Thomas S Jacques, David M Mirsky, Matthew T Whitehead, Maha S Zaki, Tipu Sultan, Pasquale Striano, Anna C Jansen, Maarten Lequin, Linda S de Vries, Mariasavina Severino, Andrew C Edmondson, Lara Menzies, Philippe M Campeau, Henry Houlden, Amy McTague, Stephanie Efthymiou, Kshitij Mankad

**Affiliations:** Developmental Biology and Cancer Section, University College London Great Ormond Street Institute of Child Health, London WC1N 1EH, UK; Department of Neuroradiology, Great Ormond Street Hospital for Children NHS Foundation Trust, London WC1N 3JH, UK; Department of Neuroradiology, Great Ormond Street Hospital for Children NHS Foundation Trust, London WC1N 3JH, UK; Unit of Functional Neurosurgery, National Hospital for Neurology and Neurosurgery, London WC1N 3BG, UK; Department of Neuromuscular Disorders, University College London Queen Square Institute of Neurology, London WC1N 3BG, UK; Department of Neuromuscular Disorders, University College London Queen Square Institute of Neurology, London WC1N 3BG, UK; Division of Human Genetics, Children’s Hospital of Philadelphia, Philadelphia, PA 19104, USA; Department of Paediatric Neurology, The Children’s Hospital and the University of Child Health Sciences, Lahore, Punjab 54000, Pakistan; Department of Clinical Genetics, Human Genetics and Genome Research Institute, National Research Centre, Dokki, Cairo 12622, Egypt; Department of Paediatric Neurology, Children’s Hospital Colorado, University of Colorado School of Medicine, Aurora, CO 80045, USA; Department of Neurosciences, Rehabilitation, Ophthalmology, Genetics, Maternal and Child Health, University of Genova and IRCCS Istituto Giannina Gaslini, 16147 Genova, Italy; Department of Paediatric Neurology, University Hospital of Pisa, 56126 Pisa, Italy; Childhood and Adolescence Neurology and Psychiatry Unit, ASST GOM Niguarda, Health Sciences Department, Università degli Studi di Milano, 20142 Milano, Italy; Department of Neurosciences, Rehabilitation, Ophthalmology, Genetics, Maternal and Child Health, University of Genova and IRCCS Istituto Giannina Gaslini, 16147 Genova, Italy; Childhood and Adolescence Neurology and Psychiatry Unit, ASST GOM Niguarda, Health Sciences Department, Università degli Studi di Milano, 20142 Milano, Italy; Department of Clinical Genetics, Antwerp University Hospital, University of Antwerp, 2650 Edegem, Belgium; Department of Paediatric Neurology, Antwerp University Hospital, University of Antwerp, 2650 Edegem, Belgium; Department of Paediatric Neurology, Antwerp University Hospital, University of Antwerp, 2650 Edegem, Belgium; Department of Neuroradiology, Vall d’Hebron University Hospital, 08035 Barcelona, Spain; Department of Paediatric Neurology, Vall d’Hebron University Hospital, 08035 Barcelona, Spain; Department of Neurology, Faculty of Medicine, Assiut University, Assiut 71515, Egypt; Department of Paediatrics, Wah Medical College NUMS, Wah Cantonment, Punjab 47000, Pakistan; Department of Paediatric Neurology and Development, Dr M.R. Khan Shishu Hospital and Institute of Child Health, Dhaka 1216, Bangladesh; Department of Paediatric Neurology and Development, Dr M.R. Khan Shishu Hospital and Institute of Child Health, Dhaka 1216, Bangladesh; Department of Paediatrics, The Children’s Hospital and the University of Child Health Sciences, Lahore, Punjab 54000, Pakistan; Department of Paediatrics, The Children’s Hospital and the University of Child Health Sciences, Lahore, Punjab 54000, Pakistan; Department of Paediatrics, The Children’s Hospital and the University of Child Health Sciences, Lahore, Punjab 54000, Pakistan; Division of Human Genetics, Children’s Hospital of Philadelphia, Philadelphia, PA 19104, USA; Department of Neuromuscular Disorders, University College London Queen Square Institute of Neurology, London WC1N 3BG, UK; Department of Neuromuscular Disorders, University College London Queen Square Institute of Neurology, London WC1N 3BG, UK; Department of Paediatric Radiology, Texas Children’s Hospital, Baylor College of Medicine, Houston, Houston, TX 77030, USA; Department of Radiology and Imaging Sciences, Indiana University School of Medicine, Indianapolis, IN 46202, USA; Department of Radiology, Boston Children’s Hospital, Harvard Medical School, Boston, MA 02115, USA; Department of Clinical Genetics, University Medical Centre Utrecht, 3584 CX Utrecht, The Netherlands; Oxford Centre for Genomic Medicine, Oxford University Hospitals NHS Foundation Trust, Oxford OX3 7HE, UK; Laboratory of Immunoglycobiology, Research Institute for Microbial Diseases, Osaka University, Osaka 565, Japan; Department of Neuroradiology, Great Ormond Street Hospital for Children NHS Foundation Trust, London WC1N 3JH, UK; Department of Neuroradiology, Great Ormond Street Hospital for Children NHS Foundation Trust, London WC1N 3JH, UK; Department of Clinical Genetics, Great Ormond Street Hospital for Children NHS Foundation Trust, London WC1N 3JH, UK; Department of Clinical Genetics, Great Ormond Street Hospital for Children NHS Foundation Trust, London WC1N 3JH, UK; Department of Clinical Genetics, Great Ormond Street Hospital for Children NHS Foundation Trust, London WC1N 3JH, UK; Department of Neurology, Great Ormond Street Hospital for Children NHS Foundation Trust, London WC1N 3JH, UK; Developmental Biology and Cancer Section, University College London Great Ormond Street Institute of Child Health, London WC1N 1EH, UK; Department of Histopathology, Great Ormond Street Hospital for Children NHS Foundation Trust, London WC1N 3JH, UK; Department of Neuroradiology, Children’s Hospital Colorado, University of Colorado School of Medicine, Aurora, CO 80045, USA; Division of Neuroradiology, Children’s Hospital of Philadelphia, Philadelphia, PA 19104, USA; Perelman School of Medicine, University of Pennsylvania, Philadelphia, PA 19104, USA; Department of Clinical Genetics, Human Genetics and Genome Research Institute, National Research Centre, Dokki, Cairo 12622, Egypt; Department of Paediatric Neurology, The Children’s Hospital and the University of Child Health Sciences, Lahore, Punjab 54000, Pakistan; Department of Neurosciences, Rehabilitation, Ophthalmology, Genetics, Maternal and Child Health, University of Genova and IRCCS Istituto Giannina Gaslini, 16147 Genova, Italy; Department of Paediatric Neurology, Antwerp University Hospital, University of Antwerp, 2650 Edegem, Belgium; Department of Radiology and Nuclear Medicine, University Medical Centre Utrecht, 3584 CX Utrecht, The Netherlands; Department of Neonatology, University Medical Centre Utrecht, 3584 CX Utrecht, The Netherlands; Neuroradiology Unit, IRCCS Istituto Giannina Gaslini, 16147 Genova, Italy; Division of Human Genetics, Children’s Hospital of Philadelphia, Philadelphia, PA 19104, USA; Perelman School of Medicine, University of Pennsylvania, Philadelphia, PA 19104, USA; Department of Clinical Genetics, Great Ormond Street Hospital for Children NHS Foundation Trust, London WC1N 3JH, UK; Department of Paediatrics, CHU Sainte Justine Research Centre, University of Montreal, Montreal QC H3T 1C5, Canada; Department of Neuromuscular Disorders, University College London Queen Square Institute of Neurology, London WC1N 3BG, UK; Department of Neurology, Great Ormond Street Hospital for Children NHS Foundation Trust, London WC1N 3JH, UK; Developmental Neurosciences, University College London Great Ormond Street Institute of Child Health, London, WC1N 1EH, UK; Department of Neuromuscular Disorders, University College London Queen Square Institute of Neurology, London WC1N 3BG, UK; Developmental Biology and Cancer Section, University College London Great Ormond Street Institute of Child Health, London WC1N 1EH, UK; Department of Neuroradiology, Great Ormond Street Hospital for Children NHS Foundation Trust, London WC1N 3JH, UK

**Keywords:** congenital disorders of glycosylation, developmental delay, epilepsy, GPI, neurodevelopmental disorder, neuroimaging

## Abstract

Inherited glycosylphosphatidylinositol deficiency disorders (IGDs) are a group of rare multisystem disorders arising from pathogenic variants in glycosylphosphatidylinositol anchor pathway (GPI-AP) genes. Despite associating 24 of at least 31 GPI-AP genes with human neurogenetic disease, prior reports are limited to single genes without consideration of the GPI-AP as a whole and with limited natural history data.

In this multinational retrospective observational study, we systematically analyse the molecular spectrum, phenotypic characteristics and natural history of 83 individuals from 75 unique families with IGDs, including 70 newly reported individuals; the largest single cohort to date.

Core clinical features were developmental delay or intellectual disability (DD/ID, 90%), seizures (83%), hypotonia (72%) and motor symptoms (64%). Prognostic and biologically significant neuroimaging features included cerebral atrophy (75%), cerebellar atrophy (60%), callosal anomalies (57%) and symmetric restricted diffusion of the central tegmental tracts (60%). Sixty-one individuals had multisystem involvement including gastrointestinal (66%), cardiac (19%) and renal (14%) anomalies. Though dysmorphic features were appreciated in 82%, no single dysmorphic feature had a prevalence >30%, indicating substantial phenotypic heterogeneity. Follow-up data were available for all individuals, 15 of whom were deceased at the time of writing. Median age at seizure onset was 6 months. Individuals with variants in synthesis stage genes of the GPI-AP exhibited a significantly shorter time to seizure onset than individuals with variants in transamidase and remodelling stage genes of the GPI-AP (*P* = 0.046). Forty individuals had intractable epilepsy. The majority of individuals experienced delayed or absent speech (95%), motor delay with non-ambulance (64%), and severe-to-profound DD/ID (59%). Individuals with a developmental epileptic encephalopathy (51%) were at greater risk of intractable epilepsy (*P* = 0.003), non-ambulance (*P* = 0.035), ongoing enteral feeds (*P* < 0.001) and cortical visual impairment (*P* = 0.007). Serial neuroimaging showed progressive cerebral volume loss in 87.5% and progressive cerebellar atrophy in 70.8%, indicating a neurodegenerative process. Genetic analyses identified 93 unique variants (106 total), including 22 novel variants. Exploratory analyses of genotype-phenotype correlations using unsupervised hierarchical clustering identified novel genotypic predictors of clinical phenotype and long-term outcome with meaningful implications for management.

In summary, we expand both the mild and severe phenotypic extremities of the IGDs, provide insights into their neurological basis, and vitally, enable meaningful genetic counselling for affected individuals and their families.

## Teaser

Sidpra *et al.* systematically delineate the clinical, molecular, and natural history spectrum of 83 individuals with inherited glycosylphosphatidylinositol deficiency disorders. They define core clinical and genetic features predictive of disease severity, with implications for management and genetic counselling.

## Introduction

Conserved in eukaryotes, the glycosylphosphatidylinositol anchor pathway (GPI-AP) is integral to the post-translational modification of numerous proteins vital for cell signalling and fundamental to early human neurogenesis and neural development.^1-6^ Inherited glycosylphosphatidylinositol deficiency disorders (IGDs) are a group of rare, seemingly heterogenous, multisystem disorders typically arising from biallelic variants in GPI-AP genes and characterized by early-onset seizures, hypotonia and neurodevelopmental delay.^7-9^ To date, 24 of at least 31 GPI-AP genes have been associated with disease in humans, rendering IGDs responsible for ∼0.15% of all neurodevelopmental disorders.^10-34^ However, despite the established principle that different pathogenic variants in the same gene may result in different clinical phenotypes, whilst pathogenic variants in different genes of the same pathway can result in similar clinical phenotypes, the current literature is limited to explorations of single genes without consideration of the GPI-AP as a whole and with very limited natural history data.^7,35-37^ As such, the full clinical and molecular spectrum of the IGDs has not yet been investigated.

Here, we systematically analyse the molecular characteristics, phenotypic spectrum and long-term clinical outcomes of 83 individuals from 75 unique families with genetically-confirmed or clinically diagnosed IGDs. By showing that IGDs have a broad phenotypic spectrum, ranging from mild motor impairment and normal cognition to spastic quadriplegia and profound intellectual disability with early death, we delineate a core set of clinical and imaging features with prognostic implications. We further identify novel genotypic determinants of clinical phenotype and patient outcome with meaningful implications for management, surveillance and genetic counselling. Finally, we take an integrated clinical, imaging and molecular approach to provide novel biological insights, further establishing the central role of GPI-anchored proteins in normal human brain development.

## Materials and methods

### Study design and patient ascertainment

This multinational retrospective observational study aimed to characterize the clinical phenotype, molecular characteristics and natural history of individuals with IGDs. Site-specific ethical approval was obtained from all institutions prior to commencement. Written informed consent was obtained for all individuals whose data are presented at an individual, rather than aggregate, level and for individuals reported with accompanying clinical photography and/or videography, in accordance with the Declaration of Helsinki.^38^ Data are reported in line with the Strengthening Reporting of Observational Studies in Epidemiology (STROBE) statement (Supplementary material).^39^

Individuals were recruited from 16 centres in 10 countries via established international collaborations and the Queen Square Genomics reference network. Inclusion criteria were: (i) genetically or clinically diagnosed IGD, i.e. identification of a monoallelic *PIGA* variant in hemizygotic males or of biallelic variants (one homozygous or two compound heterozygous) in all other GPI-AP genes in an individual with a consistent clinical phenotype; and (ii) sufficient clinical data available for interrogation. Individuals with co-variants in genes outside of the GPI-AP were excluded.

### Molecular testing

Variants in GPI-AP genes were identified by next-generation whole exome sequencing (67.6%), DNA panel sequencing (23.0%) or whole genome sequencing (9.5%) at certified genetic laboratories. Segregation analysis was performed by Sanger sequencing. The damaging effect of 11 variants in eight individuals was confirmed using flow cytometry (Supplementary material, ‘Methods’ section). All variants were harmonized with the Ensembl canonical transcript of the gene of interest from the GRCh38/hg38 human reference genome build and reinterpreted by an independent board-certified clinical geneticist (V.S.) in consultation with the referring clinician and in line with standardized criteria: namely, the American College of Medical Genetics and Genomics (ACMG) and Association for Molecular Pathology (AMP) guidelines or the Association for Clinical Genomic Science (ACGS) guidelines, as per national guidance governing the referring institution.^40,41^ Individuals with pathogenic or likely pathogenic variants in GPI-AP genes consistent with the mode of inheritance and with a supportive clinical phenotype were classified as genetically-confirmed. If no other variants were identified, individuals with variants of unknown significance (VUS) in GPI-AP genes consistent with the mode of inheritance and with a highly sensitive clinical phenotype were classified as clinically diagnosed. Prior to inclusion, individuals with VUS underwent further, independent review by a second board-certified clinical geneticist (P.C.) to ensure consensus regarding variant plausibility. To minimize potential bias resulting from the inclusion of individuals with VUS, subset analyses were performed to compare the phenotype of genetically-confirmed and clinically diagnosed individuals. This pragmatic approach has been validated in prospective multinational trials given the need for both increased next-generation sequencing in infantile-onset epilepsy and transparent variant reporting with the view to future reclassification.^42-47^ Detailed descriptions of testing methodology and inclusion rationale are provided in the Supplementary material ‘Sheet Two’. Variants were visualized using ProteinPaint.^48^

### Clinical characterization

The electronic medical record was retrospectively interrogated by the recruiting clinician for clinical, biochemical, radiological and genetic data using a comprehensive, standardized proforma.

Core clinical features were defined as having >50% prevalence across all reported individuals. The frequency at which clinical characteristics are reported was compared to the last systematic review of IGDs.^7^ Dysmorphic features were described by the recruiting clinician in accordance with recommendations from Elements of Morphology.^49^ Clinical photography was centrally reviewed, if available. Other phenotypic information was collected using standardized Human Phenotype Ontology (HPO) terms.^50^

Developmental delay (DD) was defined as mild, moderate, severe or profound in individuals aged ≤5 years; individuals aged >6 years were defined as having mild, moderate, severe or profound intellectual disability (ID).^51^ Seizure type was classified in accordance with the International League Against Epilepsy (ILAE) 2017 criteria.^52^ Epilepsy syndromes were classified in accordance with the ILAE 2022 criteria.^53,54^ Individuals with evidence of dual developmental and epileptic activity contributing to impaired neurocognitive function were diagnosed with a developmental and epileptic encephalopathy (DEE); individuals with co-existing DD/ID and epilepsy but no evidence of epileptic activity contributing to impaired neurocognitive function were diagnosed with a developmental encephalopathy (D + E).^53,55-57^ Seizure control was defined using ILAE 2010 criteria: in brief, complete seizure freedom for at least three times the maximum pre-intervention inter-seizure interval (for seizures within the preceding 12 months) or 12 months, whichever period was longer.^58^ Ambulatory status was assessed using a modified World Health Organisation (WHO) motor developmental milestones classification.^59,60^ EEGs, EMGs and skeletal radiographs were centrally reviewed, if available.

### Neuroimaging acquisition and review

Clinically acquired MRI of the brain was reviewed in consensus by an independent panel of three board-certified paediatric neuroradiologists (S.S., A.B., K.M.) in concert with the referring clinician. Due to the number of participating centres and the retrospective nature of the study, there was significant heterogeneity in terms of scanner manufacturer, magnet field strength, sequences acquired and imaging parameters. Minimum MRI brain sequences for inclusion were sagittal T_1_-weighted and axial T_2_-weighted, both with slice thickness ≤5 mm. Additional sequences, including T_2_-weighted FLAIR, susceptibility-weighed imaging (SWI), gradient recalled-echo (GRE), diffusion-weighted imaging (DWI) and diffusion tensor imaging (DTI) were reviewed, if available. In 15 individuals, for whom 28 MRIs were available as raw Digital Imaging and Communications in Medicine (DICOM) files at the host institution, further quantitative analyses of callosal and cerebellar biometry were performed to verify the accuracy of consensus qualitative assessments.^61-63^ Definitions of pertinent neuroanatomical structures and pathological findings are presented in the Supplementary material, ‘Methods’ section.

### Regional gene expression plots

Gene expression maps were constructed with custom Python code using publicly available microarray expression data from the Allen Human Brain Atlas (AHBA).^64,65^ Full methods are provided in the Supplementary material, ‘Methods’ section.

### Statistical analyses

Statistical analyses were performed using Python version 3.12.1 (Python Software Foundation, Wilmington, Delaware, USA) and R version 4.3.2 (R Foundation for Statistical Computing, Vienna, Austria).

The normality of continuous variables was tested using histogram visualization and the Shapiro-Wilk test. Normally distributed continuous variables are reported as the mean ± standard deviation (SD) and compared using Student's *t*-test. Non-normally distributed continuous variables are reported as the median and interquartile range (IQR). Categoric variables are reported descriptively as percentage frequencies and compared using the chi-square or Fisher's exact test, as appropriate. Time-to-event data were modelled using the Kaplan-Meier estimator and differences between groups evaluated using the log rank test. Exploratory analyses of genotype-phenotype correlations were performed using scaled Euclidian unsupervised hierarchical clustering and plotted as a heat map with dendrogram linkage.^66^ Sample sizes are reported for each analysis. Missing data were encountered at random and corresponding patients were discarded from subsequent, associated statistical analyses. In all instances, hypotheses were two-tailed and *P* < 0.05 was considered statistically significant.

## Results

### Demographic information

**Figure 1:**
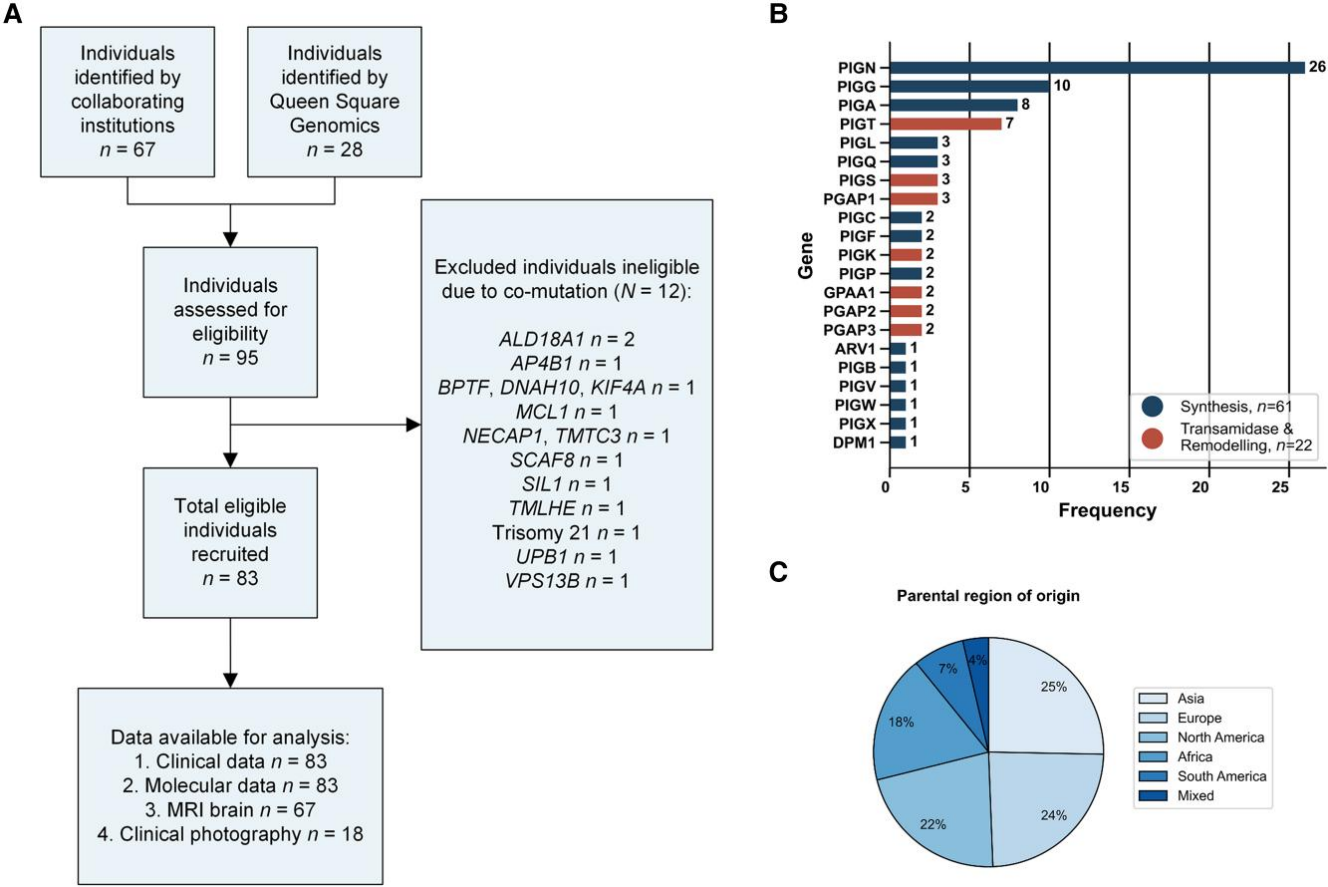
**Study flow chart and cohort characteristics.** (**A**) Study flow chart detailing individuals available for analysis and individuals excluded. (**B**) A total of 83 individuals enrolled in the study, the majority of whom have biallelic variants in *PIGN*, *PIGG*, *PIGA* and *PIGT*. Raw frequencies are printed on the respective bars. (**C**) Enrolled individuals show good coverage of all major regions with the majority being of Asian or European descent. Percentage frequencies are presented on the respective segments.

This study included 83 individuals from 75 unique families with homozygous or compound heterozygous variants in GPI-AP genes; 12 individuals were excluded due to co-variants in non-GPI-AP genes (Fig. 1A). Seventy individuals are newly reported; all previously published individuals (*n* = 13) are reported with new and follow-up data. Seventy-four individuals had genetically-confirmed IGDs (i.e. pathogenic or likely pathogenic variants in GPI-AP genes). Nine individuals had a clinically diagnosed IGD (i.e. VUS in GPI-AP genes), two of which were compound heterozygous with a pathogenic or likely pathogenic variant.

Individuals were of differing ethnic backgrounds and originated from 21 countries including, but not limited to, regions with a high prevalence of consanguinity (Fig. 1C). Consanguinity was reported in 26 families (31.3%), though this varied across genes. Eight families (10.7%) had more than one affected individual. No association with other neurological disease was found upon assessment of two to four generation family history. Genetic pedigrees are provided in Supplementary Fig. 1.

The median age at first clinical presentation was 4.9 months (IQR 2.0–11.8 months) whilst the median age at last clinical follow-up was 4.0 years (IQR 2.2–8.5 years). The male-to-female ratio was 0.8:1.0. The most common GPI-AP genes included in our cohort were *PIGN* (31.3%), *PIGG* (12.0%), *PIGA* (9.6%) and *PIGT* (8.4%) (Fig. 1B).

### Obstetric history

Most children were born at term (72/83; 86.7%), with a median gestational age of 39 weeks (IQR 37.3–40 weeks). Twenty-seven children (32.5%) had obstetric complications: eight (9.64%) had polyhydramnios; six (7.2%) required instrumented delivery; four (4.8%) required emergency caesarean section; three (3.6%) had premature rupture of the membranes; two (2.4%) had decreased foetal movements requiring intervention; and seven (8.4%) required ventilatory support and were admitted to neonatal intensive care. Anthropometric data were available as follows: birth weight (46/67), birth height (32/67), birth head circumference (28/67). Mean birth weight was 3.25 kg ± 0.55 kg; median birth height was in the 51st centile; median birth head circumference was in the 35th centile.

### Phenotypic spectrum

**Figure 2:**
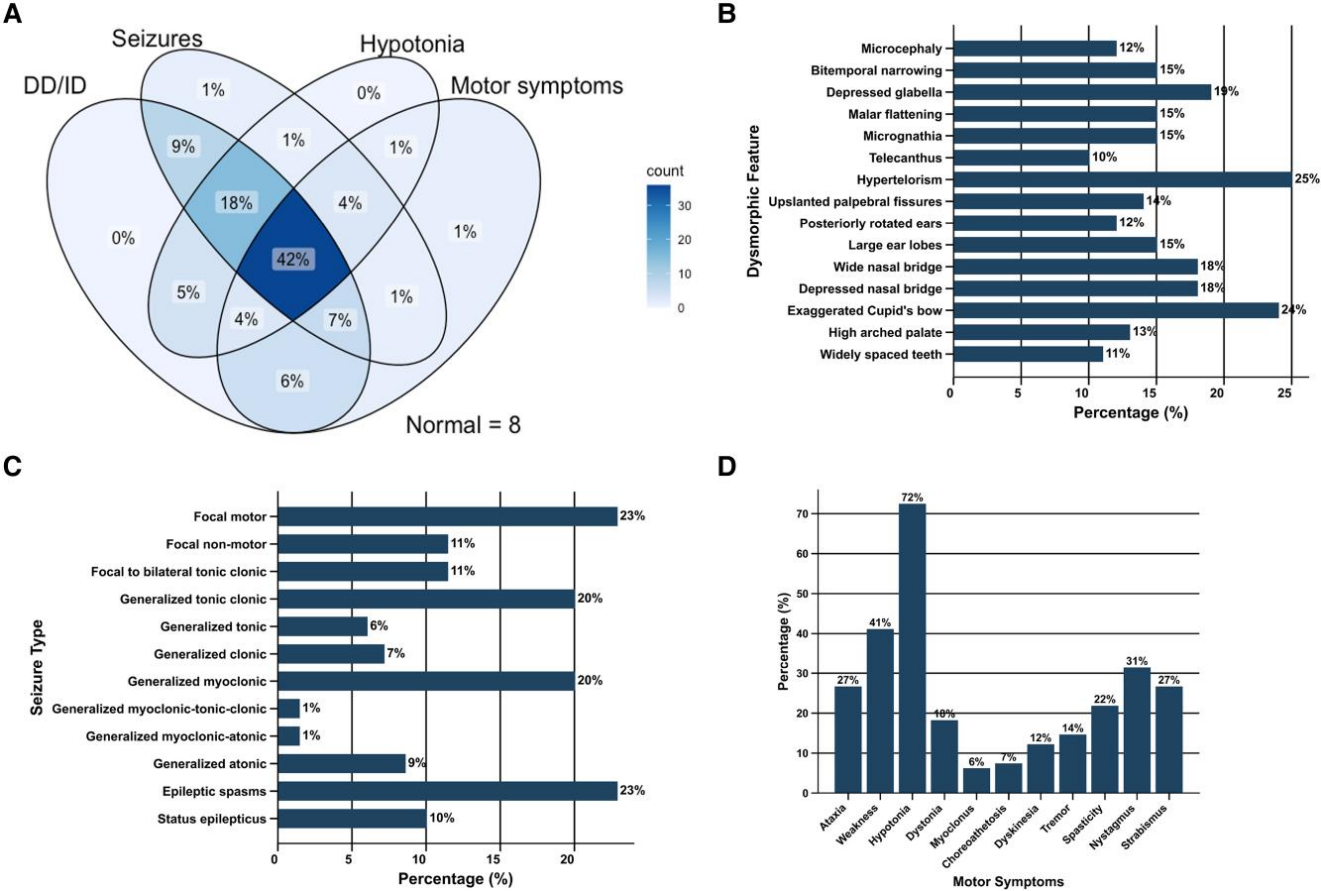
**Defining clinical features of the IGDs.** (**A**) Venn diagram of core neurological features (DD/ID, seizures, hypotonia and motor symptoms) shows some sensitivity, with all core symptoms clustering in 42% of affected individuals. (**B**) Bar chart of dysmorphic features with prevalence >10% across the cohort. The vast majority of features affect <25% of individuals, indicating substantial phenotypic heterogeneity. (**C**) Bar chart of ILAE seizure types shows that the majority of affected individuals had seizures of generalized onset despite similarly evident heterogeneity. (**D**) Bar chart of motor symptoms shows core features and further characterises the hyperkinetic spectrum of disordered movement seen in individuals with IGDs, with a subset of individuals exhibiting cerebellar and extrapyramidal signs. In all bar charts, percentage frequencies are presented on the respective bars. DD = developmental delay; ID = intellectual disability; IGD = inherited glycosylphosphatidylinositol deficiency disorders; ILAE = International League Against Epilepsy.

**Figure 3:**
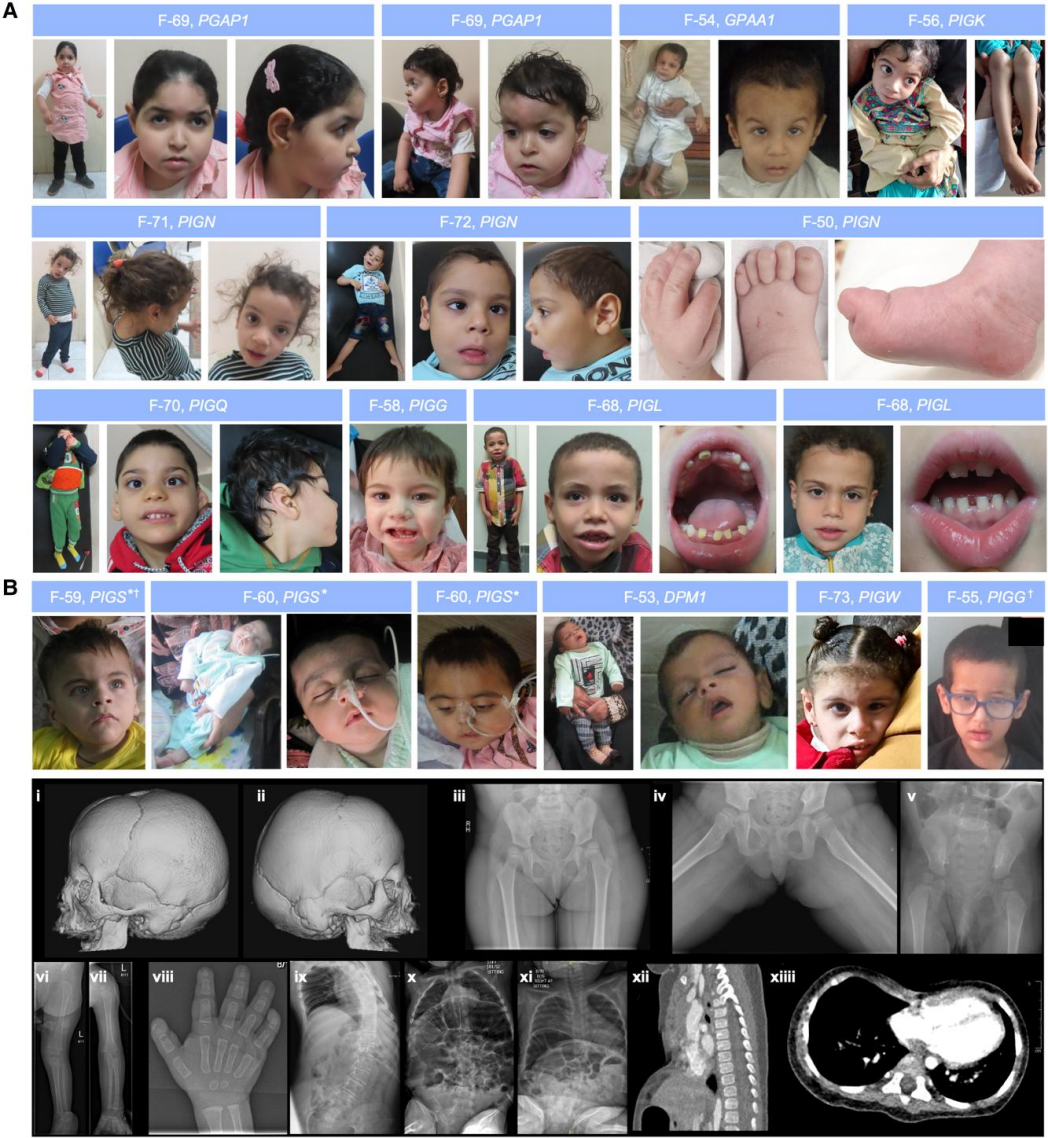
**Dysmorphic features and skeletal findings in individuals with IGDs.** (**A**) Clinical photography of individuals with IGDs demonstrates the broad dysmorphic spectrum, with substantial phenotypic heterogeneity. Individual-level annotation for these children is provided in the Supplementary material, ‘Sheet 3’. (**B**) Skeletal radiographs of individuals with IGDs. (**i** and **ii**) 3D surface-rendered CT of the head shows asymmetric bicoronal synostosis resulting in a brachycephalic head shape in an individual with *PIGN*-IGD aged 11 months. (**iii**–**v**) Developmental dysplasia of the hip in two individuals with *PIGT*-IGD (**iii** and **iv**) and *PIGB*-IGD (**v**), respectively. An anteroposterior (AP, **iii**) radiograph shows subluxation of the left and right hips with shallow acetabula and ∼50% and 25% lateral uncovering, respectively. Enlocation is seen on the frog-leg view (**iv**). (**v**) AP radiograph shows slender iliac bones, wide ischiopubic synchondroses and subluxation of the femoral heads, with an abnormally rounded appearance. (**vi**–**viii**) Further skeletal findings in the same individual with *PIGB*-IGD. AP radiographs of the left upper and lower limbs (**vi** and **vii**) show mildly slender long bones, whilst an AP radiograph of the right hand (**viii**) shows phalangeal tuft hypoplasia in digits two to four, central osteolysis of distal phalanx one, and distal aphalangia of digit five. (**ix**–**xi**) Scoliosis in three individuals with *PIGT*-IGD (**ix**), *ARV1*-IGD (**x**) and *PIGA*-IGD (**xi**), respectively. All images are AP thoracic radiographs. (**ix**) A levoconvex thoracic scoliosis centred on T10 with a Cobb angle of 62°. (**x**) A similar C-shaped levoconvex scoliosis with a Cobb angle of ∼23°. (**xi**) A whole spine levoconvex curve centred on the thoracolumbar junction. (**xii** and **xiii**) Midsagittal (**xii**) and axial (**xiii**) thoracic CT of the same individual with *PIGA*-IGD as in **xi** shows pectus excavatum with significant narrowing of the AP chest diameter (Haller index equal to four) and a bifid right fourth rib (not shown). (**xii**) An exaggerated whole spine kyphosis with loss of normal lumbar lordosis. IGD = inherited glycosylphosphatidylinositol deficiency disorders. *Reproduced with permission from Efthymiou *et al*.^67^^†^Clinical video available.

The core clinical features exhibited were DD/ID (75/83; 90.4%), seizures (69/83; 83.1%), hypotonia (60/83; 72.3%) and motor symptoms (53/83; 63.9%). All core clinical features were found together in 42.2% of individuals (Fig. 2A). Exemplar clinical photography of children with hypotonia in the context of IGDs is provided in Fig. 3A and Supplementary Video 1.^67^ Apart from hypotonia (72.3%) and muscle weakness (41.0%), motor symptoms typically manifested as hyperkinetic disordered movement (53/83; 63.9%), as summarized in Fig. 2D. Ataxia of gait was observed in 22 individuals (26.5%). An exemplar clinical video of a child with ataxia in the context of *PIGG*-IGD is provided in Supplementary Video 2. No sex differences were identified (Supplementary Fig. 2).

DEE was diagnosed in 50.7% (35/69) of individuals with seizures whilst D + E was diagnosed in 46.4% (32/69); two individuals (2.4%), both with pathogenic variants in *PIGG*, had epilepsy with normal development (Patients F-46 and F-58). Of the 14 individuals who did not experience seizures, 12 had DD/ID, ranging from mild to profound. Age at seizure onset was not significantly different in individuals with DEE and D + E (*P* = 0.135). Though most individuals were not diagnosed with other epilepsy syndromes, three (3.6%) were diagnosed with Lennox-Gastaut syndrome and two were diagnosed with infantile epileptic spasms syndrome (IESS; 2.4%). Seizure type was significantly heterogenous (Fig. 2C). The most common seizure types were focal motor (16/69; 23.2%), epileptic spasms (16/69; 23.2%), generalized tonic clonic (14/69; 20.3%) and generalized myoclonic (14/69; 20.3%). Seven individuals (10.1%) experienced status epilepticus. No differences in seizure type were seen in individuals with DEE compared with D + E. EEGs were available for review in 62 individuals (74.7%). Though no specific EEG features were identified, findings were in keeping with a DEE or D + E with focal and/or generalized epilepsy. Generalized epileptiform discharges were observed in 30 individuals (48.4%) with spike waves in a further 21.0% (13/62) and sharp waves in 3.2% (2/62). Focal epileptiform discharges were observed in nine individuals (14.5%). Twenty-two individuals (35.5%) had generalized slowing with absence of normal background rhythms. Hypsarrhythmia was seen in five individuals (8.1%), three with variants in *PIGA* and two siblings with variants in *PIGN*. Periodic complexes were seen in one individual. Twelve individuals (19.4%) had a normal EEG.

EMG was performed in nine individuals (10.8%) and was normal in four. Five individuals exhibited signs of a diffuse motor neuropathy involving the lumbar and bulbar regions (3/9; 33.3%) or of an axonal sensorimotor neuropathy (2/9; 22.2%). CSF was tested in 29 individuals (34.9%): in isolation, two had high protein, one had hyperglycorrhachia and one had low folate. All other CSF parameters were normal.

Dysmorphic facial features were appreciated in 69 individuals (83.1%). No single dysmorphic feature had a prevalence of >30%, indicating substantial phenotypic heterogeneity. Dysmorphic features with a prevalence of >10% are shown in Fig. 2B. A comprehensive summary of all dysmorphic features is provided in Supplementary Table 1. Musculoskeletal anomalies were found in 31 individuals (37.3%) and included: scoliosis (22/83; 26.5%), developmental dysplasia of the hip (8/83; 9.6%), joint hypermobility (6/83; 7.2%), pectus excavatum (5/83; 6.0%), short arthrogrypotic limbs (4/83; 4.8%) and pectus carinatum (1/83; 1.2%). Figure 3 exhibits the spectrum of dysmorphic features and skeletal findings in individuals with IGDs.

Sixty individuals (72.3%) had multisystem involvement. Most commonly, individuals had gastrointestinal involvement (55/83; 66.3%), with gastro-oesophageal reflux present in 43.4% (36/83) and 47.0% being at risk of aspiration (39/83). Nineteen individuals (22.9%) suffered from constipation, 9.0% had hypertriglyceridaemia (6/67) and 7.2% were truncally obese (6/83). One individual with a likely pathogenic variant in *PIGA* had a congenital diaphragmatic hernia (Patient F-16). Cardiac disease was identified in 16 individuals (19.3%) and typically manifested as a septal defect (13/83; 15.7%%), including: three individuals with a patent foramen ovale, four individuals with a ventricular-septal defect, two individuals with a patent ductus arteriosus, two individuals with a patent foramen ovale and an atrial-septal defect, one individual with a patent ductus arteriosus and a ventricular-septal defect, and one individual with an atrial-septal defect and a ventricular-septal defect. Mitral prolapse and aortic root dilatation were seen separately in two individuals. No individuals were diagnosed with a cardiomyopathy or had conductive anomalies. Fourteen individuals (16.9%) had renal involvement including hydronephrosis (12/83; 14.5%), renal cysts (4/83; 4.8%) and renal dysplasia (3/83; 3.6%). Urinary calcium was normal in 93.3% of individuals tested (56/60).

Serum biochemistry was normal in most individuals. Of note, plasma phosphate was high in only 9.7% of individuals tested (6/62) and low in 4.8% (3/62). Plasma alkaline phosphatase (ALP) was also normal in 66.2% of individuals tested (45/68), high in 25.0% (17/68) and low in 8.8% (6/68). Full details on serum ALP results including genetic associations are provided in Supplementary Table 2. Further subset analysis of individuals with high ALP showed no significant association with clinical outcome. Serum transferrin was normal in 22 tested individuals; transferrin isoelectric focussing was also normal in 19 tested individuals. Three individuals had normal total serum *N*-glycans whilst one individual (Patient F-23) had a slightly raised Man5/9 ratio. Serum calcium, thyroid and parathyroid, immunologic and haematologic function were grossly normal.

### Neuroimaging characteristics

Sixty-seven (80.7%) individuals underwent brain MRI; 41 (61.2%) at 3 T and 26 (38.8%) at 1.5 T. The median age at first scan was 10.1 months (IQR 4.1–32.6 months) whilst the median age at last scan was 2.4 years (IQR 1.3–6.4 years). Of these individuals, 24 (35.8%) were scanned at multiple time points and 52 (77.6%) were scanned with DWI.

**Figure 4:**
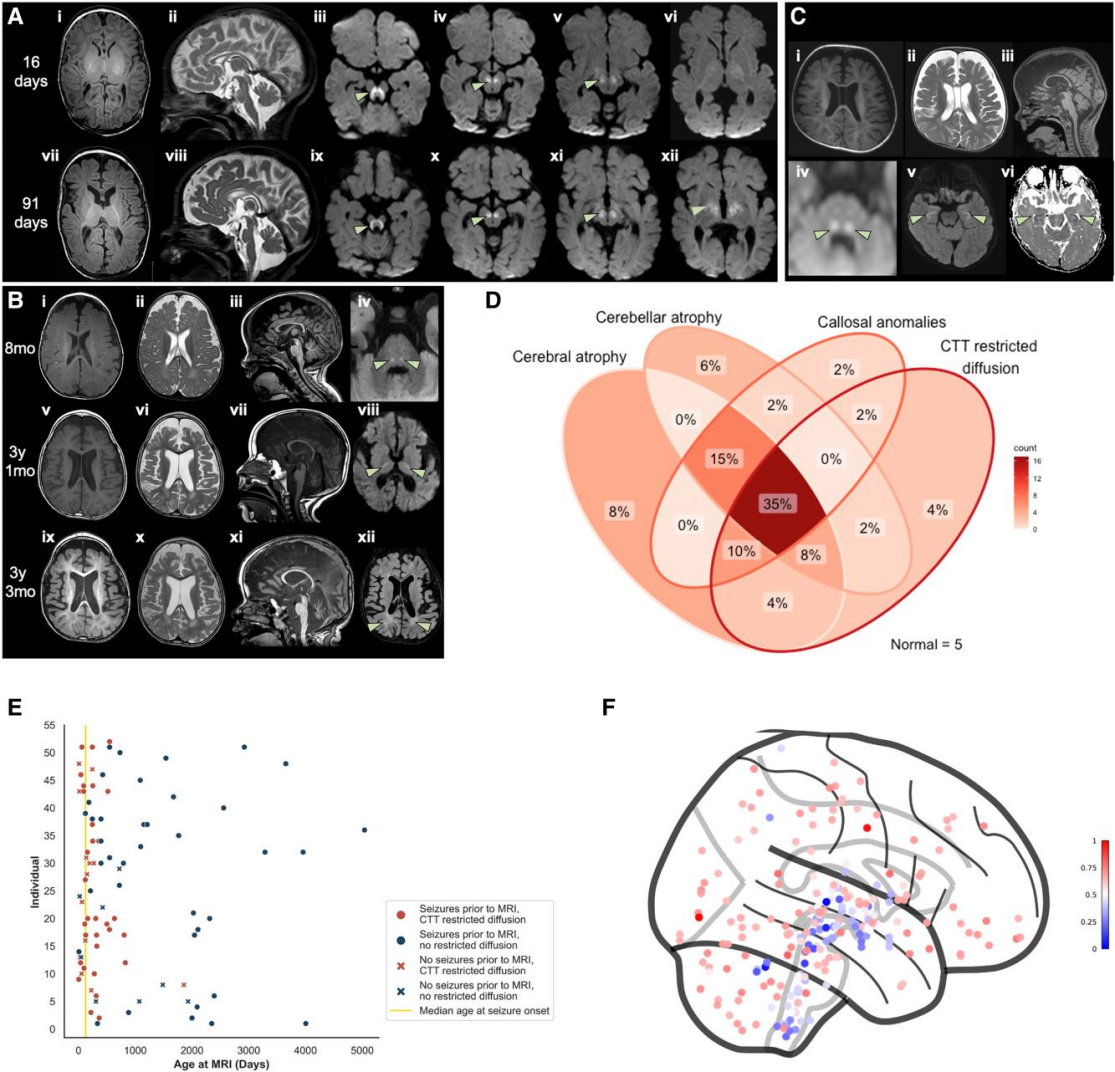
**Neuroimaging findings in individuals with IGDs.** (**A**) Sequential brain MRIs of a child with *PIGN*-IGD aged 16 days (**i**–**vi**) and 91 days (**vii**–**xii**). Initially, there is thinning of the callosal body with down-sloping of the splenium (**ii**, sagittal T_2_-weighted), a small pons and restricted diffusion of the central tegmental tracts, superior cerebellar peduncles, globus pallidus internus, subthalamic nucleus, substantia nigra, posterior limb of the internal capsule and ventral thalamus (arrow in **iii**–**vi**, axial diffusion-weighted). On follow-up imaging, rapid and progressive frontotemporal-predominant cerebral atrophy (**vii**, axial T_1_-weighted) and anterior-predominant cerebellar vermian atrophy (**viii**, sagittal T_2_-weighted) are seen. The restricted diffusion is noted to exhibit some resolution caudally but becomes more prominent rostrally (arrow in **ix**–**xii**, axial diffusion-weighted). (**B**) Sequential brain MRIs of a second child with *PIGN*-IGD aged 8 months (mo) (**i**–**iv**), 3 years 1 month (**v**–**viii**) and 3 years 3 months (**ix**–**xii**). Neuroimaging at presentation shows frontal volume loss (**i**, axial T_1_-weighted and **ii**, axial T_2_-weighted), thinning of the callosal body with a down-sloping splenium (**iii**, sagittal T_1_-weighted), anterior-predominant cerebellar vermian atrophy (**iii**, sagittal T_1_-weighted) and restricted diffusion of the central tegmental tracts and superior cerebellar peduncles (arrows in **iv**, axial diffusion-weighted). Follow-up MRIs show progressive frontotemporal volume loss (**v**, **vi**, **ix** and **x**, axial T_1_- and T_2_-weighted), progressive cerebellar atrophy (**vii** and **xi**, sagittal T_1_-weighted), rostral ascent of the restricted diffusion to involve the posterior limb of the internal capsule (arrows in **viii**, axial diffusion-weighted) and a diffuse periventricular leukodystrophy (arrows in **xii**, axial T_2_ fluid-attenuated inversion recovery). A transient mesial temporal diffusion abnormality at 3 years and 1 month of age (not shown) was likely seizure-related. Note the trigonocephalic head shape with metopic synostosis. (**C**) Brain MRI of a child with *PIGA*-IGD (Patient PIGA-1) aged 2 months (**i**–**iv**) shows frontal volume loss (**i**, axial T_1_-weighted and **ii**, axial T_2_-weighted), thinning of the callosal body and down-sloping of the splenium (**iii**, sagittal T_1_-weighted) and more subtle restricted diffusion of the central tegmental tracts (arrows in **iv**, axial diffusion-weighted). Hippocampal atrophy is also noted (arrows in **v**, axial T_1_-weighted, and **vi**, axial diffusion-weighted). (**D**) Venn diagram of core neuroimaging findings in individuals with complete MRI: cerebral atrophy, cerebellar atrophy, callosal anomalies and restricted diffusion of the central tegmental tracts (CTT). No significant differences were seen in individuals for whom diffusion-weighted imaging (DWI) was not performed for financial reasons. (**E**) Scatter plot of all individuals with DWI showing the onset and temporal resolution of central tegmental tract restricted diffusion independent of epileptic activity. In particular, note that diffusion changes are seen even in individuals with no seizures prior to neuroimaging. (**F**) Regional expression pattern of all GPI-AP genes in healthy controls derived from normative AHBA data shows physiologically clustered reduced expression in the brainstem and deep grey nuclei (blue). This pattern of expression did not vary between genes or between the synthesis and transamidase and remodelling components of the GPI-AP (Supplementary Fig. 3). AHBA = Allen Human Brain Atlas; GPI-AP = glycosylphosphatidylinositol anchor pathway; IGD = inherited glycosylphosphatidylinositol deficiency disorders.

The most prevalent findings on brain MRI were cerebral atrophy (50/67; 74.6%), cerebellar atrophy (40/67; 59.7%), callosal anomalies (38/67; 56.7%) and symmetric restricted diffusion of the central tegmental tracts (31/52; 59.6%) (Fig. 4A–C). All core neuroimaging features were found together in 35% of individuals (Fig. 4D). Cerebral atrophy typically exhibited a frontotemporal predominance (40/50; 80%), though 10 individuals (20%) presented with global cerebral atrophy. Cerebellar atrophy demonstrated an anterior-posterior gradient, with the anterior lobule being most severely affected in 67.5% (27/40). Nine individuals with anterior-predominant cerebellar atrophy demonstrated a bright anterosuperior cerebellar cortex on T_2_-weighted FLAIR imaging. Of individuals with callosal anomalies, 55.3% (21/38) displayed thinning of the callosal body and down-sloping of the splenium, 34.2% (13/38) exhibited thinning of the callosal body only and 10.5% (4/38) exhibited down-sloping of the splenium only. Quantitative analyses confirmed a small cerebellar size and thin corpus callosum under the third centile respective to age for all individuals in whom this qualitative evaluation was made by the neuroimaging review panel. Though symmetric restriction of the central tegmental tracts was seen in all individuals with diffusion changes, symmetric restricted diffusion also extended to the globi pallidi (16/52; 30.8%), superior cerebellar peduncles (14/52; 28.8%), internal capsule (9/52; 17.3%), subthalamic nuclei (5/52; 9.6%), thalami (4/52; 7.7%), optic radiations (4/52; 7.7%), hypothalamus (1/52; 1.9%) and corticospinal tracts (1/52; 1.9%). Restricted diffusion of the whole dentatorubrothalamocrotical tract was seen in 12 individuals (23.1%). The pattern of cerebral and cerebellar atrophy in individuals with IGDs mimics the pattern of high regional GPI-AP gene expression seen in AHBA data whilst the pattern of restricted diffusion seen in individuals with IGDs mimics the pattern of relatively low regional GPI-AP gene expression throughout the brainstem and deep grey nuclei (Fig. 4F and Supplementary Fig. 3).

Hippocampal atrophy (Fig. 3C) was seen in 19.4% (13/67), all of whom had seizures prior to their first MRI. A diffuse leukodystrophy pattern (Fig. 3B) was seen in 16.4% (11/67), all but two of whom were over 2 years of age at the time of imaging (median 2.8 years; IQR 2.0–4.0 years); delayed myelination was seen in 11.9% (8/67). The anterior commissure was small in 7.5% (5/67) whilst 4.5% (3/67) had underdeveloped olfactory bulbs and small optic nerves. One individual (Patient F-33), with pathogenic variants in *PIGQ*, had perisylvian polymicrogyria (Supplementary Fig. 4). Six (9.0%) individuals had further incidental findings: Blake's pouch cyst (2/67), Rathke's cleft cyst (2/67), developmental venous anomaly of the left frontal lobe (1/67), middle cranial fossa arachnoid cyst (1/67), and congenital interhypothalamic adhesion (1/67). Five individuals, all with variants in *PIGN* or *PIGG*, had normal MRI brains and mild-to-profound DD/ID.

Craniosynostosis was confirmed on neuroimaging in 16.4% (11/67). The most commonly fused sutures were the metopic (3/11), sagittal (3/11) and bicoronal (3/11). The metopic and sagittal sutures were both fused in 2/11 individuals. Fusion of the metopic suture was confirmed with metopic ridging and trigonocephaly in all individuals.^68^ A further six individuals were noted to have positional plagiocephaly (7.1%).

Serial neuroimaging showed progressive, frontotemporal-predominant volume loss in all but three individuals (21/24; 87.5%), which progressed to global brain atrophy in 33.3% (8/24). Two individuals developed bilateral frontal subdural collections secondary to rapid volume loss. Progressive cerebellar atrophy was seen in 70.8% (17/24; Supplementary Fig. 5) with pontine atrophy in a further 10.4% (7/67). Together, these findings are indicative of a neurodegenerative process. Restricted diffusion of the central tegmental tracts typically resolved on follow-up imaging (18/24; 75.0%), as demonstrated in Fig. 4E, and ascended rostrally with age (15/24; 62.5%).

Supplementary Table 3 details correlations between the core neuroimaging features of IGDs and clinical phenotype. Cerebral volume loss was significantly associated with severe-to-profound DD/ID (*P* = 0.046) and hypotonia (*P* = 0.020). Non-ambulant individuals typically exhibited cerebral volume loss (*P* < 0.001), cerebellar atrophy (*P* = 0.036) and callosal anomalies (*P* = 0.034). Hyperkinesia was associated with all core neuroimaging features (*P* < 0.001). Seizures were less common in individuals with callosal anomalies (*P* = 0.035). No significant associations were found between ataxia of gait and neuroimaging features.

### Natural history

Follow-up data were available for all individuals. The median duration of follow-up from first clinical presentation was 2.9 years (IQR 1.4–6.6 years). The oldest individual (Patient F-1), with compound heterozygous pathogenic variants in *PIGT*, was aged 20 years at last clinical follow-up.

**Figure 5:**
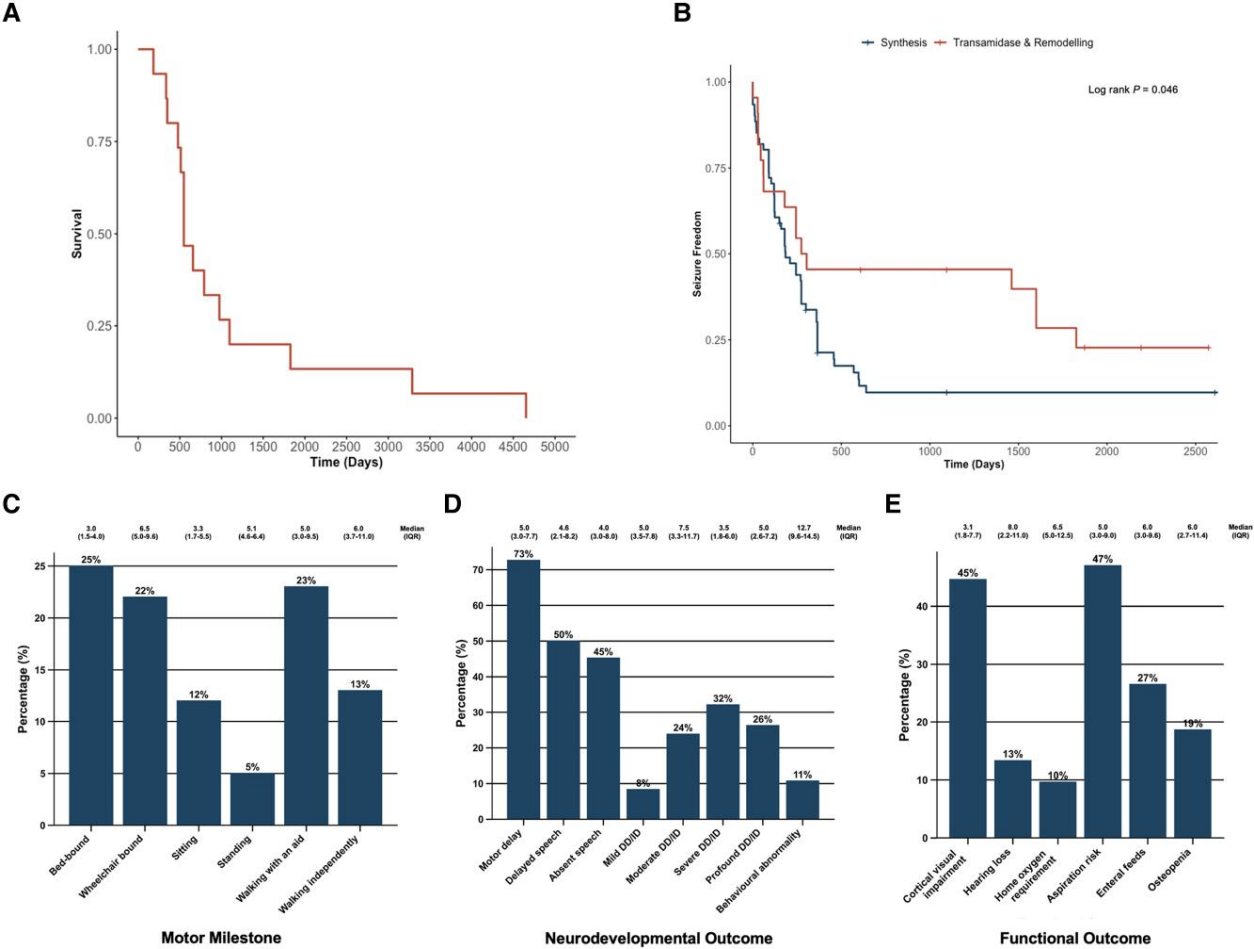
**Natural history of individuals with IGDs.** (**A**) Kaplan-Meier curve of all-cause mortality across our cohort, *n* = 15. (**B**) Kaplan-Meier curve of seizure freedom shows a significantly earlier age at seizure onset for individuals with variants in synthesis stage genes of the GPI-AP when compared to individuals with variants in transamidase and remodelling stage genes of the GPI-AP (log rank *P* = 0.046). (**C**) Bar chart of motor milestones shows that the majority of individuals were non-ambulant at last clinical follow-up but that outcomes ranged from normal independent walking to spastic quadriplegia. (**D**) Bar chart of neurodevelopmental outcomes at last clinical follow-up shows DD/ID, speech delay and motor delay as almost universal features. (**E**) Bar chart of functional outcomes exhibits a high prevalence of cortical visual impairment and significant non-neurological morbidity at last clinical follow-up, including complex nutritional requirements. In all bar charts, percentage frequencies are printed on the respective bars and the median age at last clinical follow-up is printed above each bar with the interquartile range bracketed. DD = developmental delay; GPI-AP = glycosylphosphatidylinositol anchor pathway; ID = intellectual disability; IGDs = inherited glycosylphosphatidylinositol deficiency disorders.

Fifteen individuals (18.1%) were deceased at the time of writing. Median survival for these individuals was 1.5 years (IQR 1.4–2.8 years) (Fig. 5A). The most common cause of death was respiratory failure secondary to recurrent respiratory tract infection (5/15). Seizures also accounted for five deaths: three individuals experienced post-ictal decompensated cardiorespiratory failure, whilst two had intractable status epilepticus. Four individuals had sudden unexpected deaths in epilepsy (SUDEP). One individual died due to gastrointestinal complications. There was no association between genetic variant and cause of death.

Median age at seizure onset was 5.9 months (IQR 2.0–10.0 months). Individuals with variants in synthesis stage genes of the GPI-AP exhibited a significantly shorter time to seizure onset (median 5.6 months; IQR 3.0–9.9 months) than individuals with variants in transamidase and remodelling stage genes of the GPI-AP (median 7.0 months; IQR 1.4–19.5 months); log rank *P* = 0.046 (Fig. 5B). Forty individuals (57.1%) experienced intractable, drug-resistant epilepsy with ongoing seizures. Individuals with D + E were significantly more likely to achieve seizure control (*P* = 0.003) and seizure control on monotherapy (*P* = 0.010) than those with DEE. Though no single anti-seizure medication was effective in the majority of individuals to whom it was prescribed, levetiracetam achieved seizure control in 38.5% of individuals (15/39) as part of a polytherapeutic regimen. Pyridoxine was trialled at variable doses in 22 individuals (26.5%), achieving complete seizure control in four individuals and partial control in three individuals. In one individual, a trial of pyridoxine paradoxically increased seizure frequency. No association was found between pyridoxine dose and seizure control. No individuals underwent epilepsy surgery.

Motor milestones, neurodevelopmental and functional outcomes were variably achieved and a significant proportion of individuals experienced non-neurological disability (Fig. 5C–E). The majority of individuals experienced delayed or absent speech (79/83; 95.2%), motor delay with non-ambulance (53/83; 63.9%) and severe-to-profound DD/ID (49/83; 59.0%). Long-term clinical outcomes were significantly worse in individuals with a DEE than those with a D + E: individuals with a DEE were more likely to be non-ambulant (*P* = 0.035), require ongoing enteral feeds (*P* < 0.001) or have cortical visual impairment (*P* = 0.007). Nine individuals had behavioural abnormalities including autism spectrum disorder (ASD, 4/9), attention deficit hyperactivity disorder (ADHD, 2/9), and mixed ASD and ADHD (3/9). Developmental regression was observed in five older individuals with D + E who had a median age at last follow-up of 6 years; individuals with DEE had more severe presentations and consistently missed developmental milestones whereas the milder D + E phenotype appeared to be more permissive of development, with some individuals reaching normal developmental milestones—albeit with regression in a minority.

Bone density was tested in 59 individuals using dual emission or energy X-ray absorptiometry (DEXA) scanning, 11 of whom were osteopenic (18.6%) and one was osteoporotic (1.7%). Two individuals with pathogenic variants in *PIGG* and *PGAP2* (Patients F-3 and F-6, respectively) experienced precocious puberty with dysmenorrhoea and adrenarche, characterized by pubic and maxillary hair growth and body odour but no breast development.

### Molecular spectrum

A detailed characterization of all variants including allele frequencies, *in silico* predictions of damaging effects and ACMG-AMP/ACGS classification is provided in the Supplementary material, ‘Sheet Two’.

We identified 93 unique variants (106 total), including 22 novel variants. Biallelic variants were homozygous in 36 families and compound heterozygous in 31 families. Hemizygous variants in *PIGA* were present in eight families. Forty-two unique variants were classified as pathogenic and 40 unique variants were classified as likely pathogenic; 11 unique variants were classified as of unknown significance, two of which were compound heterozygous with a pathogenic or likely pathogenic variant. The pathogenicity of 11 variants in eight individuals, including four novel variants, was confirmed using flow cytometry (Supplementary material, ‘Results’ section and Supplementary Fig. 9). The majority of variants were missense (52/93; 55.9%) or predicted to be splice-altering (17/93; 18.3%). Protein truncating variants, predicted to cause loss-of-function via nonsense-mediated mRNA decay, had a frequency of 19.4% (18/93), including 10 frameshift variants and eight nonsense variants. Eight microdeletions, two large deletions and one copy number loss were also seen across 12 families. There was one identified insertion (*PGAP1* c.2357_2358insTA), which occurred *in trans* with c.393dup; one deleterious in frame variant (*PIGN* c.133_135delAGA) which occurred *in trans* with the splice region variant c.1434+5G>A; one homozygous in frame deletion (*PIGL* c.347_355del); and one hemizygous start loss variant (*PIGA* c.1A>G). Recurrent missense variants were identified in *PIGG* (c.1515G>A, three unrelated individuals); *PIGL* (c.175C>T, two unrelated individuals); *PIGN* (c.1694G>T, four unrelated individuals; c.932T>G, three unrelated individuals); and *PIGT* (c.1079G>T, two unrelated individuals and two siblings). One individual with homozygous variants in *PIGX* (c.4G>T p.Ala2Ser) is reported, establishing *PIGX* as a potential candidate gene, which requires further interrogation and independent verification, ideally with functional studies. Full phenotypic characteristics for this individual (Patient F-67) and individuals with recurrent variants are available in the Supplementary material ‘Results’ section.

All variants had very low allele frequencies across multiple variant frequency databases, with total allele counts ranging from 0–1677/1 500 000 (Supplementary material, ‘Sheet 2’). *In silico* analyses predicted high conservation of affected amino acid residues and deleteriousness of the respective genomic changes in most individuals (Supplementary material, ‘Sheet 2’ and Supplementary Fig. 6). Visualization of variants in genes with more than five affected individuals showed a broad distribution across primary protein structures without mutational hotspots. Visualization of genes with novel variants is provided in Supplementary Fig. 7.

### Genotype–phenotype correlations

**Figure 6:**
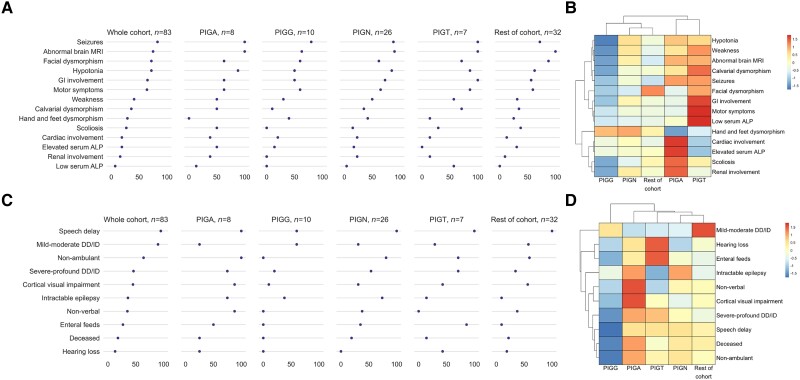
**IGDs show meaningful phenotypic and natural history variability when clustered by genotype.** (**A**) Dot plots of genotypic groups show differential frequencies of core phenotypic features. (**B**) Exploratory unsupervised hierarchical clustering of genotype-phenotype correlations shows aggregation and dendrogram linkage of genotypic groups based on the presence of core clinical features. (**C**) Dot plots of genotypic groups show differential frequencies of long-term clinical outcomes. (**D**) Exploratory unsupervised hierarchical clustering of genotype-outcome correlations shows aggregation and dendrogram linkage of genotypic groups based on long-term clinical outcomes. In **B** and **D**, the colour scale represents scaled, relative frequencies—i.e. dark blue is low frequency relative to the other genes and dark red is high frequency relative to the other genes. DD = developmental delay; ID = intellectual disability; IGD = inherited glycosylphosphatidylinositol deficiency disorders.

Exploratory analyses of genotype–phenotype correlations using unsupervised hierarchical clustering revealed meaningful differences in phenotype and outcome (Fig. 6B and D with relative percentage frequencies shown in Fig. 6A and C). Importantly, we show *PIGG*-IGD results in a much milder form of IGD, being an outlier in both phenotypic and outcome clusters. Further, despite key differentials (including a very high proportion of gastrointestinal involvement, motor symptoms and low ALP in *PIGT*-IGD compared to a very high proportion of cardiac involvement, renal involvement, scoliosis and high ALP in *PIGA*-IGD), we show that both *PIGA*-IGD and *PIGT*-IGD cluster together phenotypically: characterized by the relatively high shared prevalence of seizures, hypotonia, weakness, calvarial dysmorphism and abnormal brain MRI findings in these groups. Phenotypic similarities are also shown between *PIGN*-IGD and the rest of the cohort, with few differentiating features, suggesting a milder phenotype across these groups.

Long-term clinical outcomes were also shown to be different across IGD subtypes (Fig. 6C and D): despite the similar phenotype of *PIGA*-IGD and *PIGT*-IGD, markedly different outcomes are shown. Individuals with *PIGA*-IGD are shown to have more neurological morbidity and mortality than any other group, whilst individuals with *PIGT*-IGD have more non-neurological disability. *PIGN*-IGD and *PIGA*-IGD were clustered closely given the high prevalence of intractable epilepsy within these two groups, despite *PIGN*-IGD having better outcomes across all other domains. The rest of the cohort had better outcomes than that of *PIGN*-IGD, clustering with *PIGT*-IGD, but displaying markedly less severe DD/ID and non-neurological morbidity.

No significant differences were identified in core clinical or neuroimaging features when comparing individuals with clinically diagnosed IGDs and individuals with genetically-confirmed IGDs. Protein truncating variants had no significant association with clinical outcome. In particular, there was no association with disease severity, age at symptom onset or response to anti-seizure medications. There was also no significant association between dysmorphology or seizure type and genotype.

## Discussion

We report data on the molecular spectrum, clinical phenotype and natural history of 83 individuals with IGDs—the largest single cohort to date. For the first time, we identify a core set of clinical and neuroimaging features with prognostic implications across the GPI-AP: providing evidence that individuals with hypotonia, seizures, DD/ID or motor symptoms on neurological examination and frontotemporal-predominant cerebral atrophy, cerebellar atrophy, callosal anomalies or restricted diffusion of the central tegmental tracts on brain MRI should undergo next-generation sequencing for identification of a putative genetic driver.^42^ By further defining the epileptology of the IGDs, we provide evidence that pyridoxine may be less effective at achieving seizure control than previously reported and highlight polytherapeutic regimens, including levetiracetam, as those most likely to achieve seizure control in our cohort.^69-71^ Though *PIGN*-IGD is the only IGD with recently described—albeit heterogenous—epileptology, this is expanded to include focal emotional and sensory seizures in addition to generalized myoclonic-atonic seizures.^72^ We also show the differential severity of variants in genes of the synthesis versus the transamidase and remodelling stage of the GPI-AP in time to seizure onset—providing original data to support the hypothesis of a recent review.^73^

The multisystemic phenotype of the IGDs is similarly expanded. Of particular note, our cohort has a significantly higher prevalence of calvarial dysmorphism, gastrointestinal anomalies, skeletal anomalies and brain MRI anomalies, and a significantly lower prevalence of DD/ID, nail anomalies, short fingers or hands, and elevated serum ALP than reported in the last systematic review of the IGD phenotypic spectrum (Supplementary Fig. 8).^7^ The lower prevalence of distal phalangeal hypoplasia in our cohort is clinically important given previous reports that this may represent a sensitive dysmorphic sign on examination.^74^ Similarly, though a recent review suggested that IGD-related cardiomyopathy is under-reported, no individuals in our cohort had cardiomyopathy, including individuals with complex, often *PIGA*-IGD-related, structural cardiac disease.^75^ Importantly, the significantly lower prevalence of DD/ID in our cohort expands the milder phenotypic spectrum of the IGDs: not only providing further evidence that children with IGDs can develop into adolescence with normal cognition and mild motor impairment, but also identifying novel and previously reported recurrent variants predictive of this milder phenotype with better long-term outcomes.^76^

Though the composition of our cohort is largely consistent with the relative incidence of the IGDs, with *PIGN* being the most commonly implicated gene, a potential limitation is that we do not report population-level data and risk ascertainment bias. It is, however, difficult to conclusively ascertain which IGD is the most common, and even more difficult to ascertain their relative incidence across the GPI-AP, due to the paucity of epidemiological data. Analysis of population-level data from 4293 trios enrolled in the Deciphering Developmental Disorders study identified IGDs as a cause of DD in 0.15% (*n* = 6), with the authors citing the need for larger cohorts and the fact that DD is not present in all individuals with IGDs as a confounding factor.^10,77^ This phenotypic heterogeneity renders early and accurate diagnosis of the IGDs challenging. For this reason, several biochemical biomarkers have been proposed, in particular, serum ALP—the biosynthesis of which depends on a GPI-anchoring step.^78^ Our results support reported observations that pathogenic variants in *PIGB*, *PGAP2* and *PGAP3* are associated with high serum ALP; pathogenic variants in *PIGC*, *PIGG*, *PIGK* and *GPAA1* are associated with normal serum ALP; and pathogenic variants in *PIGT* are associated with low or normal serum ALP.^79^ Notably, whilst *PIGS* is typically associated with normal serum ALP, two out of three individuals in our cohort had abnormal serum ALP. Therefore, though an abnormal serum ALP in the context of a patient with a suggestive clinical phenotype should raise clinical suspicion for an IGD, the variability in reported data and the fact that most individuals in our cohort had a normal serum ALP, highlight the fact that this cannot yet be used as a reliable diagnostic biomarker and that larger datasets are required.

Serum transferrin and total serum *N*-glycans have also been proposed as IGD biomarkers given their sensitivity for other congenital disorders of glycosylation (CDG) with *N*-glycosylation defects.^79^ Whilst clinically useful for the exclusion of these disorders, we report their limited diagnostic sensitivity for the IGDs. Similarly, transferrin isoelectric focusing—the gold-standard method for CDG screening—is shown to have poor sensitivity for the IGDs. In this light, the most sensitive biomarker for the IGDs is one of a myriad GPI-AP—including CD16, CD55, CD59 and fluorescent aerolysin (FLAER)—for which partial loss of expression on the surface of fibroblasts and granulocytes is readily assessable via routine flow cytometry.^79,80^ Though flow cytometric assessment of suspected IGDs is not typically available in routine clinical practice (with only 9.6% of our cohort tested on a research basis), it should be strongly considered, particularly for individuals with VUS in GPI-AP genes and phenotypic features of an IGD as our results show it can be a powerful contributing factor towards variant interpretation.^74^

Longitudinal neuroimaging review and exploratory computational neuroimaging analyses of AHBA data provide potential insights into the neurological basis of IGDs. It is well established that GPI-AP proteins are vital for myelination and normal white matter development.^6,80-83^ More specifically, it has been demonstrated that GPI-AP proteins are vital for the initiation and ongoing maintenance of myelination via selective association of GPI-AP proteins with glycosphingolipid-rich microdomains during oligodendrocyte maturation: targeting glycosphingolipid-rich microdomains to the myelin sheath, acting as a myelin sorting signal, and retaining adhesive contacts during the spiralling of glial processes around the axon and the laying down of the multilamellar sheath.^81^ The central tegmental tracts are one of the earliest brain regions to commence myelination, by nine postconceptional months in most individuals.^84^ Restricted diffusion of the central tegmental tracts (attributed to altered axial diffusivity) is rare in healthy individuals but has been reported in other neurometabolic disorders, cerebral palsies, epileptic syndromes (including IESS), and following vigabatrin administration.^85-92^ The prevalence of diffusion restriction in our cohort vastly exceeds all previously reported levels. Interestingly, the pattern of restricted diffusion seen in this study (Fig. 4A–C) maps to the brainstem and deep grey nuclei: the same brain areas that exhibit physiologically low expression of GPI-AP genes, as shown with normative AHBA data (Fig. 4F). We, therefore, hypothesize that individuals with IGDs are susceptible and predisposed to intramyelinic oedema in these brain areas, resulting in the reversible changes seen in our cohort on DWI.^90,93^ Despite highlighting the importance of GPI-AP proteins for normal neurodevelopment, the reversibility of the diffusion restriction and lack of significant association with core clinical features in our cohort in addition to the presence of the finding in the normal population does, however, suggest that this is a benign process with correction as the infant develops into childhood. However, given these biological insights and the relative specificity and sensitivity of the finding for the IGDs, we posit that these diffusion abnormalities represent a meaningful diagnostic neuroimaging biomarker which, in the context of an appropriate clinical history and phenotype, should prompt genetic investigation. This specificity is particularly strong in the subset of individuals with restricted diffusion of the entire dentatorubrothalamocrotical tract.

The intrinsic vulnerability of the white matter in individuals with IGDs is reflected by the high prevalence of cerebral and cerebellar atrophy in brain regions with higher physiological expression of GPI-AP genes, as shown with normative AHBA data (Fig. 4F and Supplementary Fig. 3). This correlation between the spatial severity of cortical anatomical change and differential regional gene expression has previously been shown in other neurodevelopmental disorders.^94-96^ Further, the importance of GPI-AP genes to normal myelination is reflected in the significant number of individuals in our cohort with either a diffuse leukodystrophy type neuroimaging pattern or delayed myelination. Indeed, in the first and, to our best knowledge, only published autopsy report of an individual with *PIGT*-IGD, the predominant neuropathological findings were those of substantially reduced myelination, a pronounced astrogliosis and microgliosis of the white matter, and lipid-containing macrophages in the white matter visible when stained with Oil Red O and Sudan Black—resulting in a diagnosis of an orthochromatic (sudanophilic) leukodystrophy.^97,98^ Independent of these hypotheses, the poor natural history of most individuals with IGDs, in combination with the progressively atrophic findings on brain MRI—even in the context of controlled or absent epileptic seizures—is suggestive of an underlying neurodegenerative process. These observation-driven hypotheses do, however, require validation in autopsy studies, larger cohort studies or developmental models more reflective of temporal changes in gene expression throughout the lifespan.

In summary, we take a ‘phenotype-first’ approach to characterization of the IGDs: expanding both the mild and severe phenotypic extremities; providing insights into their neurological basis; and identifying novel genotypic predictors of clinical outcome with meaningful implications for management and surveillance. Vitally, our findings enable effective and informed genetic counselling for affected individuals and their families. It is our hope that the development of this large international cohort and natural history study will permit further insights into disease pathogenesis, progression and the more precise definition of end points for future clinical trials.

## Supplementary Material

awae056_Supplementary_Data

## Data Availability

Processed data supporting the findings presented in this manuscript are available in the Supplementary material. Anonymized raw data are available from the corresponding authors upon reasonable request; sharing of individual-level clinical data beyond that reported in this manuscript may be subject to privacy restrictions and/or an appropriate data transfer agreement.
