# Supplementary material for: The clinical and genetic spectrum of inherited glycosylphosphatidylinositol deficiency disorders
Source: Brain. 2024 Mar 8;147(8):2775–90. doi: 10.1093/brain/awae056 (PMC11292905; doi:10.1093/brain/awae056)
Supplement: awae056_Supplementary_Data [file awae056_supplementary_data.zip › brain-2023-02061-File008.pdf]

Supplementary Materials

The clinical and genetic spectrum of inherited glycosylphosphatidylinositol deficiency disorders

Jai Sidpra, Sniya Sudhakar, Asthik Biswas, Flavia Massey, Valentina Turchetti, Tracy Lau, Edward Cook, Javeria Raza Alvi, Hasnaa M. Elbendary, Jerry L. Jewell, Antonella Riva, Alessandro Orsini, Aglaia Vignoli, Zara Federico, Jessica Rosenblum, An-Sofie Schoonjans, Matthias de Wachter, Ignacio Delgado Alvarez, Ana Felipe-Rucián, Nourelhoda A. Haridy, Shahzad Haider, Mashaya Zaman, Selina Banu, Najwa Anwaar, Fatima Rahman, Shazia Maqbool, Rashmi Yadav, Vincenzo Salpietro, Reza Maroofian, Rajan Patel, Rupa Radhakrishnan, Sanjay Prabhu, Klaske Lichtenbelt, Helen Stewart, Yoshiko Murakami, Ulrike Löbel, Felice D'Arco, Emma Wakeling, Wendy Jones, Eleanor Hay, Sanjay Bhate, Thomas S. Jacques, David M. Mirsky, Matthew T. Whitehead, Maha S. Zaki, Tipu Sultan, Pasquale Striano, Anna C. Jansen, Maarten Lequin, Linda S. de Vries, Mariasavina Severino, Andrew C. Edmondson, Lara Menzies, Philippe M. Campeau, Henry Houlden, Amy McTague, Stephanie Efthymiou, Kshitij Mankad

Contents

Supplementary methods

1. Neuroimaging acquisition and review
2. Regional gene expression plots
3. Flow cytometric analyses

Supplementary results: tables

1. Dysmorphic spectrum of IGDs described using terms derived from Elements of Morphology
2. Serum alkaline phosphatase (ALP) results and genetic associations
3. Core neuroimaging features and clinical-neuroradiological associations of the IGDs

Supplementary results: figures

1. Pedigrees of included families and segregation results
2. Sex difference in core clinical features
3. Regional expression of genes involved in the synthesis versus transamidase and remodelling stages of the GPI-AP
4. Perisylvian polymicrogyria in an individual with *PIGG*-IGD
5. Progressive cerebellar atrophy in an individual with *PIGG*-IGD
6. In silico variant effect prediction scores for all feasible variants
7. Lollipop plots of genes containing novel variants
8. Prevalence of core phenotypic features compared to the last systematic review of IGD phenotypic features
9. Measurement of GPI-anchored protein expression levels by flow cytometry in patient-derived granulocytes (F-8, F-50), patient-derived fibroblasts (F-23, F-24, F-29, F-34), and *PIGL*-knockout HEK293 cells (F-14)

Supplementary results: text

1. *PIGX*-IGD: clinical delineation
2. Phenotypic analysis and natural history of recurrent variants
3. Flow cytometric experiments
    a. Individuals with previously reported flow cytometric confirmation of variant pathogenicity
    b. Individuals with newly reported flow cytometric confirmation of variant pathogenicity

Supplementary Methods

Neuroimaging acquisition and review

Linear measurements and comparison to age- and sex-matched normative values is determinative of small cerebellar and callosal size. Cerebellar atrophy was further and strictly defined as enlargement of the interfolial spaces vis-à-vis folial size in a normally sized posterior fossa.[1] The central tegmental tracts (CTT) were defined as the white matter tracts running between the inferior olivary nuclei and the red nuclei.[2] The dentatorubrothalamocrotical tracts (DRTC) were defined as the white matter tracts arising in the dentate nuclei and forming the superior cerebellar peduncles prior to decussating in the midbrain, ascending via the red nuclei and ventrolateral thalamus, and projecting to widespread areas of the cerebral cortex.[3] Rather than defining hippocampal sclerosis on neuroimaging, we employ the term hippocampal atrophy, in keeping with ILAE guidance.[4]

Regional gene expression plots

Gene expression maps were constructed using publicly available microarray expression data provided by the Allen Human Brain Atlas (AHBA).[5,6] These data consist of thousands of probe samples per donor from six normative donors across the entire left hemisphere annotated by brain region, probe MRI, and Montreal Neurological Institute (MNI) coordinates. Data were pre-processed using established methods akin to those employed in the Abagen toolbox (version 0.1.4) with custom Python code.[7,8] Thirty-one genes of interest from the GPI-AP were identified within the dataset (Supplementary Sheet 1) and all other gene expression profiles discarded. To filter out noisy expression data in this subset of genes, probe samples were filtered such that any sample that did not have an expression intensity level significantly different from background was excluded within each donor, as defined by AHBA criteria (two-tailed t-test between the mean signal of probe expression and the corresponding background $P<0.01$ and the difference between the background subtracted signal and the background is significant, i.e. >2.6 standard deviations).[5] Next, sample annotations were used to match multiple samples from the same brain regions so that their average expression level could be calculated within each donor per brain region. Expression-by-brain region matrices were averaged across donors and the corresponding brain region-specific MNI coordinates provided in the original dataset. Only brain regions for which there were samples across all donors were included. Average expression data were then normalised and plotted by relative expression level in MNI space using the Nilearn toolbox.[9]

Flow cytometric analyses

Methods: F-8

50µl of blood collected in BCT CytoChex tubes was lysed with osmotic erythrocyte lysis buffer. White blood cells were incubated with a staining mixture of fluorescently labelled antibodies: CD45-PacBlue (granulocyte marker), CD55-APC, CD16b-APC- Vio770-A, CD16-ECD, and FLAER (FITC labelled aerolysin). The cells were incubated at room temperature for 25 minutes, washed twice with FACS buffer, and resuspended in FACS buffer. Flow cytometric analysis (fluorescence-activated cell sorting, FACS) was performed on a MACSQuant Analyser 10 (Miltenyi Biotec, Bergisch Gladbach, Germany). GPI marker expression was analysed on CD45 positive granulocytes with FlowJo v9.9.6 (TreeStar Inc., USA).

Methods: F-14

PIGL-knockout HEK293 cells were generated and transfected, using previously described methodology, with human strong (SRα promoter; pME) or weak TATA-box only (pTA) promoter-driven wild-type or mutant *PIGL* cDNA.[10] Two days later, the restoration of GPI-anchored protein (CD59 and DAF) surface expression was measured using FACS. The cells transfected with pME HAPIGL from were also lysed and western blotting was performed. Quantities were calculated by the band intensity normalised by GAPDH expression as a loading control and luciferase activity as a transfection efficiency.

Methods: F-23, F-24, F-29, F-34

Patient-derived fibroblast cell lines from patients with genetic mutations affecting GPI-anchor biosynthesis pathway genes and a control fibroblast cell line were assayed for cell surface expression of the GPI-anchored protein CD59 by flow cytometry. Cells were grown in low glucose (1g/L) media with 10% foetal bovine serum, 1% penicillin/streptomycin, and 1% Glutamax supplementation. Upon confluency, fibroblast cells were harvested using trypsin and washed with PBS. The fibroblasts were stained with CD59-FITC antibody (Catalog # MHCD5901, Thermo Fisher Scientific, USA) diluted 1:200 in FACS buffer (PBS, sodium azide, 2.5% FBS) on ice in the dark for 21 minutes. Cells were washed in FACS buffer and fixed using 4% Paraformaldehyde. The cells were then again washed and resuspended in FACS buffer for flow cytometry. A total of 10,000 cells were measured for fluorescence intensity of the antibody. Density plots of the fibroblast cells were gated to exclude population of debris and to select a cell population for further analysis-based on forward and side scatter (FSC-A vs. SSC-A). Single cells were gated on a diagonal (SSC-H vs. SSC-A) for all the cell lines. The cells were further gated according to the intensity of FITC emission spectrum along with SSC-H axis and the expression level of CD59-FITC was visually evaluated in the form of histogram of fluorescence intensity. The means of FITC positive cells were calculated and compared between samples. All data acquisition was done on LSRFortessa analyzer (BD Biosciences, USA) and the analysis was performed using FlowJo_10.9.0 software (TreeStar Inc., USA).

Methods: F-50

50µl of blood collected in BCT CytoChex tubes was lysed with osmotic erythrocyte lysis buffer. White blood cells were incubated with a staining mixture composed of FLAER (FITC labelled aerolysin), CD16, and CD24. The cells were incubated at room temperature for 25 minutes, washed twice with FACS buffer, and resuspended in FACS buffer. Flow cytometric analysis was performed on a FACSCanto II (BD Biosciences, USA). GPI marker expression was analysed on CD16 positive granulocytes with FlowJo v9.5.3 (TreeStar Inc., USA).

References

1. Poretti A, Boltshauser E. Terminology in morphological anomalies of the cerebellum does matter. *Cerebellum Ataxias*. 2015;2(1). doi:10.1186/S40673-015-0027-X
2. Nathan PW, Smith MC. The rubrospinal and central tegmental tracts in man. *Brain*. 1982;105(Pt 2):223-269. doi:10.1093/BRAIN/105.2.223
3. Toescu SM, Hales PW, Kaden E, Lacerda LM, Aquilina K, Clark CA. Tractographic and Microstructural Analysis of the Dentato-Rubro-Thalamo-Cortical Tracts in Children Using Diffusion MRI. *Cerebral Cortex*. 2021;31(5):2595-2609. doi:10.1093/CERCOR/BHAA377
4. Blümcke I, Thom M, Aronica E, et al. International consensus classification of hippocampal sclerosis in temporal lobe epilepsy: A Task Force report from the ILAE Commission on Diagnostic Methods. *Epilepsia*. 2013;54(7):1315-1329. doi:10.1111/EPI.12220
5. Hawrylycz MJ, Lein ES, Guillozet-Bongaarts AL, et al. An anatomically comprehensive atlas of the adult human brain transcriptome. *Nature*. 2012;489(7416):391-399. doi:10.1038/NATURE11405
6. Miller JA, Ding SL, Sunkin SM, et al. Transcriptional Landscape of the Prenatal Human Brain. *Nature*. 2014;508(7495):199. doi:10.1038/NATURE13185
7. Arnatkevičiūtė A, Fulcher BD, Fornito A. A practical guide to linking brain-wide gene expression and neuroimaging data. *Neuroimage*. 2019;189:353-367. doi:10.1016/J.NEUROIMAGE.2019.01.011
8. Romero-Garcia R, Mandal AS, Bethlehem RAI, Crespo-Facorro B, Hart MG, Suckling J. Transcriptomic and connectomic correlates of differential spatial patterning among gliomas. *Brain*. 2023;146(3):1200-1211. doi:10.1093/BRAIN/AWAC378
9. Abraham A, Pedregosa F, Eickenberg M, et al. Machine learning for neuroimaging with scikit-learn. *Front Neuroinform*. 2014;8(FEB):71792. doi:10.3389/FNINF.2014.00014/BIBTEX
10. Pagnamenta AT, Murakami Y, Taylor JM, et al. Analysis of exome data for 4293 trios suggests GPI-anchor biogenesis defects are a rare cause of developmental disorders. *Eur J Hum Genet*. 2017;25(6):669-679. doi:10.1038/EJHG.2017.32

Supplementary Results

Supplementary Table 1
Dysmorphic spectrum of IGDs described using terms derived from Elements of Morphology

**Dysmorphic feature**		**Number of individuals (*n*=83)**	**Percentage (%)**
**Skull**	Brachycephaly	5	6.0
	Dolichocephaly	5	6.0
	Macrocephaly	4	4.8
	Microcephaly	10	12.0
	Plagiocephaly	4	4.8
	Trigonocephaly	3	3.6
**Scalp hair**	Abnormally positioned hair whorl	1	1.2
	High anterior hair line	1	1.2
	Low anterior hair line	3	3.6
	Sparse scalp hair	3	3.6
	Hypopigmented hair	2	2.4
**Forehead**	Narrow forehead, bitemporal narrowing	12	14.5
	Frontal bossing	6	7.2
	Depressed glabella	16	19.3
	Prominent metopic ridge	1	1.2
**Maxilla and midface**	Malar flattening	12	14.5
	Midface retrusion, midface hypoplasia	1	1.2
	Cheekbone underdevelopment, zygomatic hypoplasia	2	2.4
**Mandible**	Broad jaw	1	1.2
	Micrognathia	12	14.5
**Periorbital**	Telecanthus	9	10.8
	Epicanthus	8	9.6
	Widely spaced eyes, hypertelorism	21	25.3
	Thick eyebrow	3	3.6
	Sparse eyebrow	6	7.1
	Highly arched eyebrow	7	8.3
	Down-slanted palpebral fissure	6	7.1
	Up-slanted palpebral fissure	12	14.3
	Short palpebral fissure	3	3.6
**Ear**	Low-set ear	4	4.8
	Increased posterior angulation of the ear, posteriorly rotated ear	10	12.0
	Crumpled ear	4	4.8
	Large lobe	12	14.5
	Posterior pit of helix	1	1.2
	Prominent helix	1	1.2
**Nose**	Wide nasal bridge, broad nasal bridge	15	18.1
	Depressed nasal bridge	15	18.1
	Anteverted nares, upturned nasal tip	7	8.4
	Broad nasal tip	2	2.4

	Short nose, small nose	4	4.8
	Bulbous nose, bulbous nasal tip	2	2.4
	Low hanging columella, broad columella	2	2.4
**Philtrum**	Deep philtrum	2	2.4
	Short philtrum	2	2.4
	Long philtrum	1	1.2
**Lips**	Exaggerated Cupid's bow	20	24.1
	Thin upper lip vermilion	5	6.0
	Tented upper lip vermilion	3	3.6
**Mouth**	Narrow mouth	2	2.4
	Broad mouth	3	3.6
**Oral cavity**	Large tongue, macroglossia	1	1.2
	High palate	11	13.3
	Submucous cleft palate	3	3.6
	Cleft uvula, bifid uvula	1	1.2
	Short hard palate	1	1.2
	Widely spaced teeth	9	10.8
**Hands and feet**	Pes planus	2	2.4
	Tapered finger	8	9.6
	Clinodactyly	5	6.0
	Cutaneous syndactyly	2	2.4
	Short distal phalanx of the finger, phalangeal hypoplasia	2	2.4
	Shortening, brachydactyly	5	6.0
	Prominent heel	1	1.2
**Nails**	Small nails, hypoplastic nails	7	8.4
	Concave nails, koilonychia	1	1.2

Supplementary Table 2
Serum alkaline phosphatase (ALP) results for all individuals tested (*n*=68)

Gene	Number of individuals reported	Number of individuals tested	Alkaline phosphatase results
ARV1	1	1	Normal serum ALP (*n*=1)
DPM1	1	1	High serum ALP (*n*=1)
GPAA1†	2	1	Normal serum ALP (*n*=1)
PGAP1	3	3	Normal serum ALP (*n*=3)
PGAP2*	2	2	High serum ALP (*n*=2)
PGAP3*	2	1	High serum ALP (*n*=1)
PIGA	8	8	High serum ALP (*n*=4)<br>Normal serum ALP (*n*=3)<br>Low serum ALP (*n*=1)
PIGB*	1	1	High serum ALP (*n*=1)
PIGC†	2	2	Normal serum ALP (*n*=2)
PIGF	2	0	NA
PIGG†	10	7	Normal serum ALP (*n*=6)<br>High serum ALP (*n*=1)
PIGK†	2	2	Normal serum ALP (*n*=2)
PIGL	3	1	High serum ALP (*n*=1)
PIGN	26	23	Normal serum ALP (*n*=18)<br>High serum ALP (n=4)<br>Low serum ALP (*n*=1)
PIGP	2	2	Normal serum ALP (*n*=1)<br>High serum ALP (n=1)
PIGQ	3	3	Normal serum ALP (*n*=3)
PIGS†	3	3	Normal serum ALP (*n*=1)<br>High serum ALP (n=1)<br>Low serum ALP (*n*=1)
PIGT^	7	7	Low serum ALP (*n*=4)<br>Normal serum ALP (*n*=3)
PIGV*	1	0	NA
PIGW	1	0	NA
PIGX	1	0	NA

* Genes, variants in which are typically associated with raised serum ALP.
† Genes, variants in which are typically associated with normal serum ALP.
^ Genes, variants in which are typically associated with normal or low serum ALP.
For all other genes, the data is less clear and requires further testing on larger patient cohorts.

References

1. Bruneel A, Cholet S, Tran NT, Mai TD, Fenaille F. CDG biochemical screening: Where do we stand?. *Biochim Biophys Acta Gen Subj*. 2020;1864(10):129652.
2. Bellai-Dussault K, Nguyen TTM, Baratang NV, Jimenez-Cruz DA, Campeau PM. Clinical variability in inherited glycosylphosphatidylinositol deficiency disorders. *Clin Genet*. 2019;95(1):112-121.

Supplementary Table 3
Core neuroimaging features and clinical-neuroradiological associations of the IGDs

		Core clinical features													
**Core neuroimaging feature**	**Number of individuals (%)**	**Seizures**	***P***	**Severe-profound intellectual disability**	***P***	**Non-ambulant**	***P***	**Hypotonia**	***P***	**Weakness**	***P***	**Ataxia**	***P***	**Hyperkinesia**	***P***
**Cerebral volume loss**	50/67 (74.6)	43/70 (61.4)	1.000	39/60 (65.0)	**0.046**	40/47 (85.1)	**<0.001**	41/50 (82.0)	**0.020**	20/25 (80.0)	0.562	10/13 (76.9)	1.000	30/38 (78.9)	**<0.001**
**Cerebellar atrophy**	40/67 (59.7)	34/70 (85%)	0.730	25/60 (41.7)	0.453	31/47 (66.0)	**0.036**	30/50 (60.0)	1.000	12/25 (48.0)	0.204	8/13 (61.5)	1.000	25/38 (65.8)	**<0.001**
**Callosal anomalies**	38/67 (56.7)	30/70 (42.8)	**0.035**	24/60 (40.0)	0.454	30/47 (63.8)	**0.034**	31/50 (62.0)	0.147	14/25 (0.56)	1.000	8/13 (61.5)	1.000	22/38 (57.9)	**<0.001**
**Restricted diffusion of the central tegmental tracts**	31/52 (59.6)	29/47 (61.7)	0.383	19/31 (61.2)	0.781	24/37 (64.9)	0.350	28/45 (62.2)	0.420	10/19 (52.6)	0.5595	7/10 (70.0)	0.721	19/31 (61.3)	**<0.001**

Supplementary Figure 1
Pedigrees of included families and segregation results

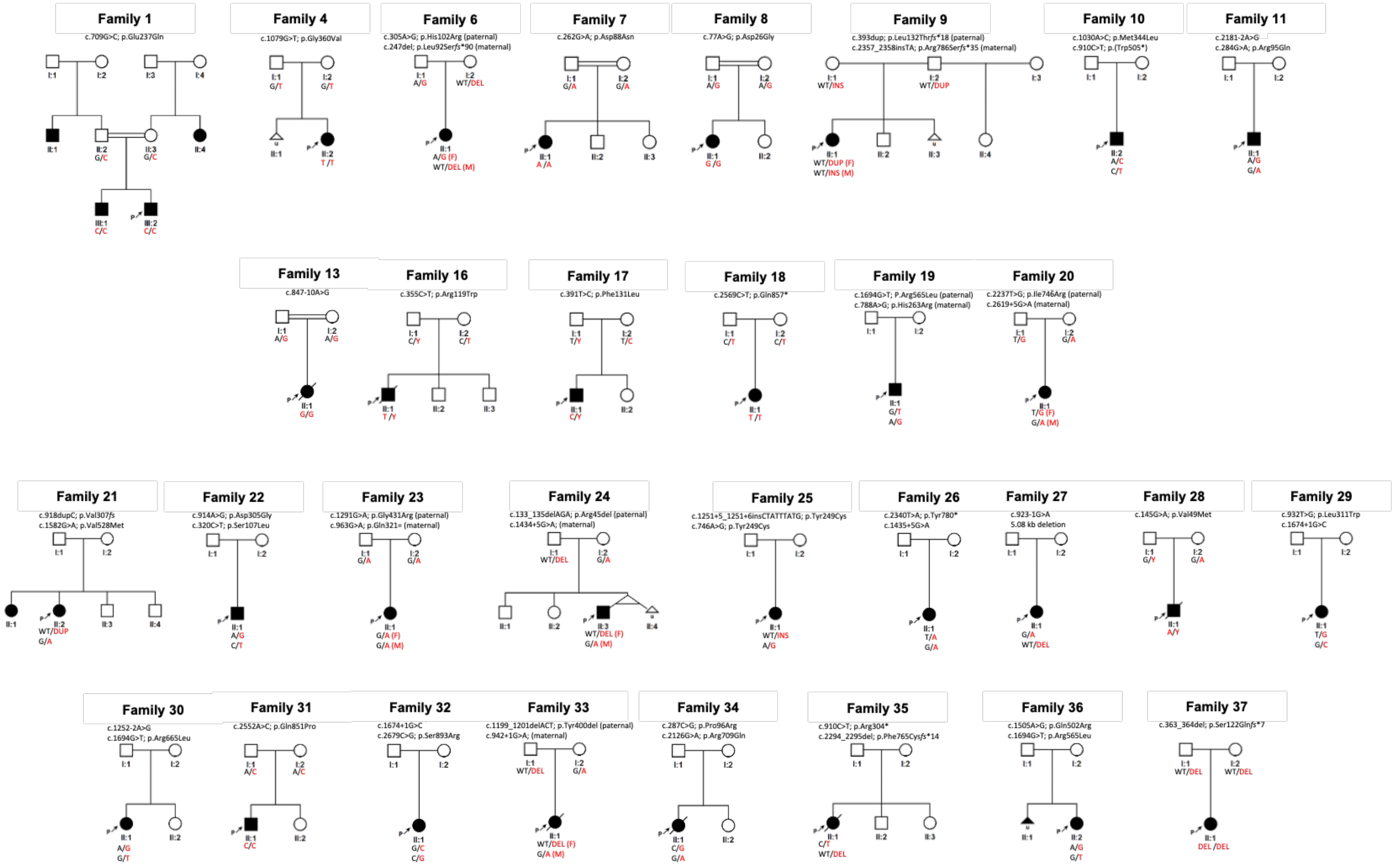

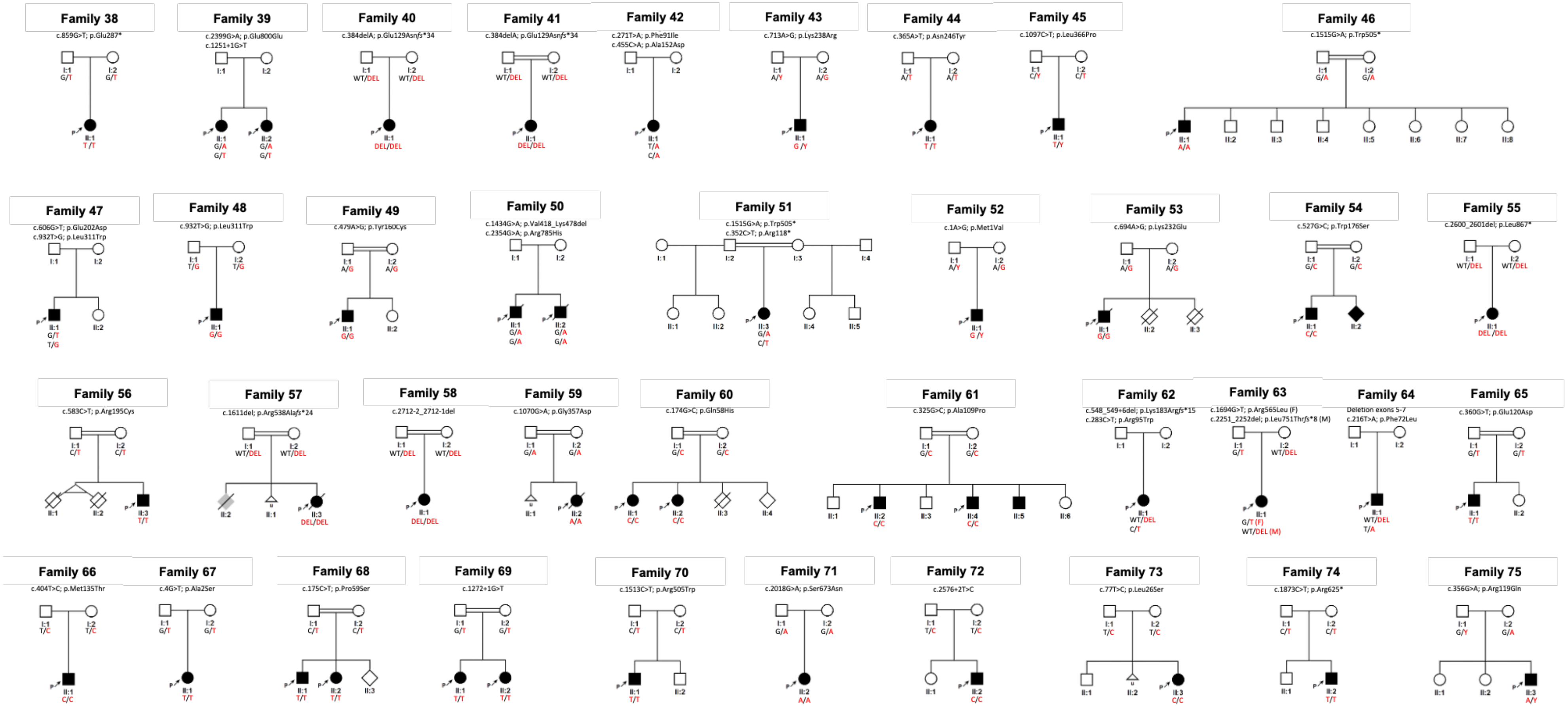
Family 38
c.859G>T; p.Glu287*
Family 39
c.2399G>A; p.Glu800Glu
c.1251+1G>T
Family 40
c.384delA; p.Glu129Asnfs*34
Family 41
c.384delA; p.Glu129Asnfs*34
Family 42
c.271T>A; p.Phe91Ile
c.455C>A; p.Ala152Asp
Family 43
c.713A>G; p.Lys238Arg
Family 44
c.365A>T; p.Asn246Tyr
Family 45
c.1097C>T; p.Leu366Pro
Family 46
c.1515G>A; p.Trp505*
Family 47
c.606G>T; p.Glu202Asp
c.932T>G; p.Leu311Trp
Family 48
c.932T>G; p.Leu311Trp
Family 49
c.479A>G; p.Tyr160Cys
Family 50
c.1434G>A; p.Val418_Lys478del
c.2354G>A; p.Arg785His
Family 51
c.1515G>A; p.Trp505*
c.352C>T; p.Arg118*
Family 52
c.1A>G; p.Met1Val
Family 53
c.694A>G; p.Lys232Glu
Family 54
c.527G>C; p.Trp176Ser
Family 55
c.2600_2601del; p.Leu867*
Family 56
c.583C>T; p.Arg195Cys
Family 57
c.1611del; p.Arg538Alafs*24
Family 58
c.2712-2_2712-1del
Family 59
c.1070G>A; p.Gly357Asp
Family 60
c.174G>C; p.Gln58His
Family 61
c.325G>C; p.Ala109Pro
Family 62
c.548_549+6del; p.Lys183Argfs*15
c.283C>T; p.Arg95Trp
Family 63
c.1694G>T; p.Arg565Leu (F)
c.2251_2252del; p.Leu751Thrfs*8 (M)
Family 64
Deletion exons 5-7
c.216T>A; p.Phe72Leu
Family 65
c.360G>T; p.Glu120Asp
Family 66
c.404T>C; p.Met135Thr
Family 67
c.4G>T; p.Ala2Ser
Family 68
c.175C>T; p.Pro59Ser
Family 69
c.1272+1G>T
Family 70
c.1513C>T; p.Arg505Trp
Family 71
c.2018G>A; p.Ser673Asn
Family 72
c.2576+2T>C
Family 73
c.77T>C; p.Leu26Ser
Family 74
c.1873C>T; p.Arg625*
Family 75
c.356G>A; p.Arg119Gln

Supplementary Figure 2
No significant sex difference in core clinical features was identified

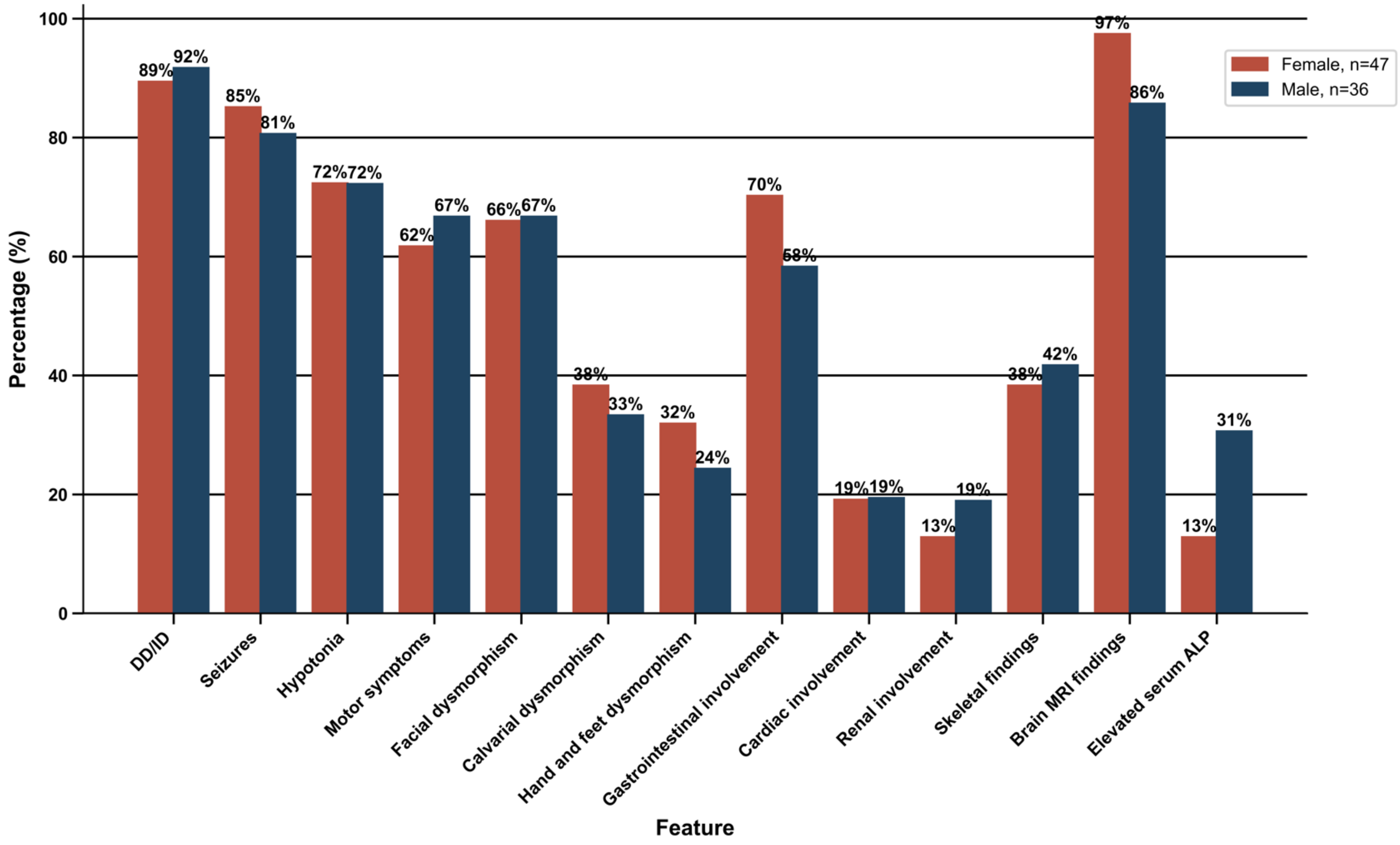

Supplementary Figure 3
Regional expression of genes involved in the synthesis (A) and transamidase and remodelling (B) stages of the GPI-AP

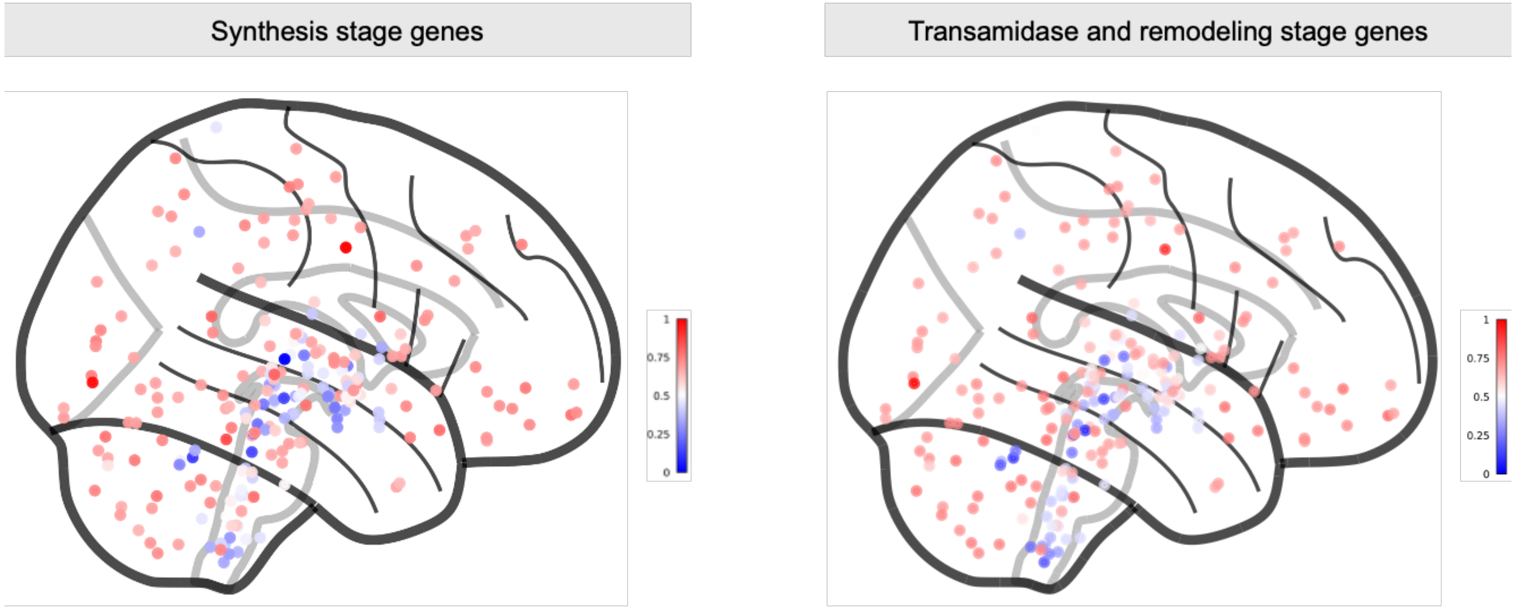

Supplementary Figure 4
Perisylvian polymicrogyria (arrow) in an individual with *PIGG*-IGD

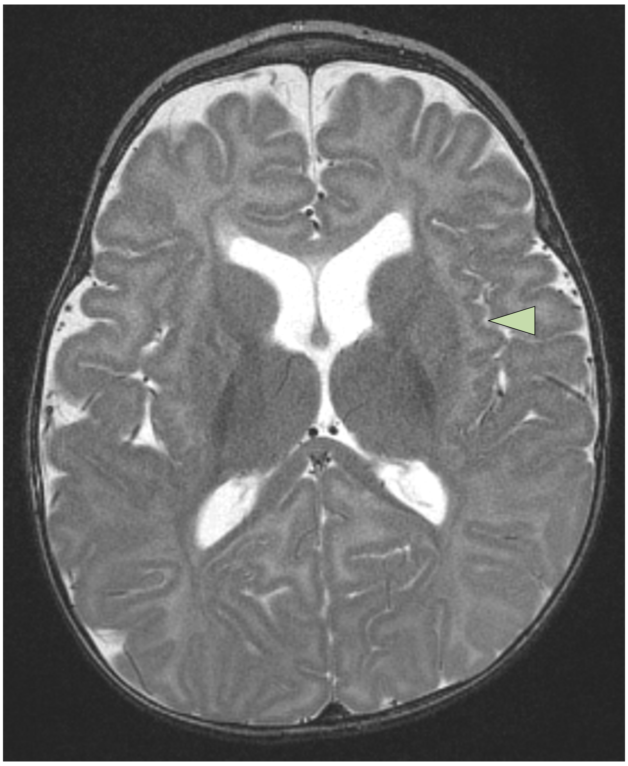

Supplementary Figure 5
Progressive cerebellar atrophy in an individual with *PIGG*-IGD

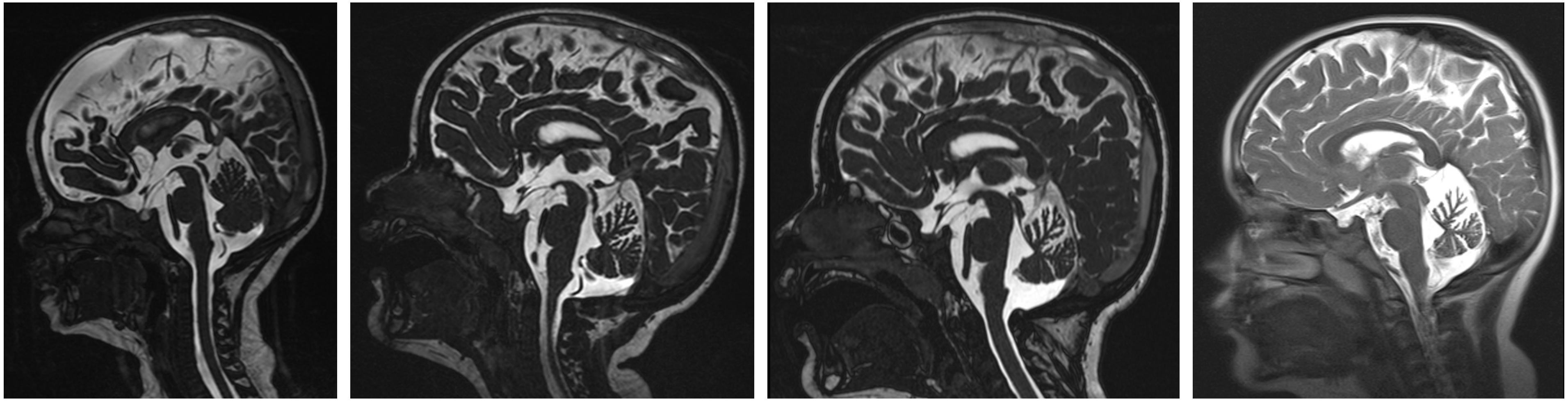

7 months 2 years 5 years 8 years

Supplementary Figure 6
***In silico* variant effect prediction scores for all feasible variants**

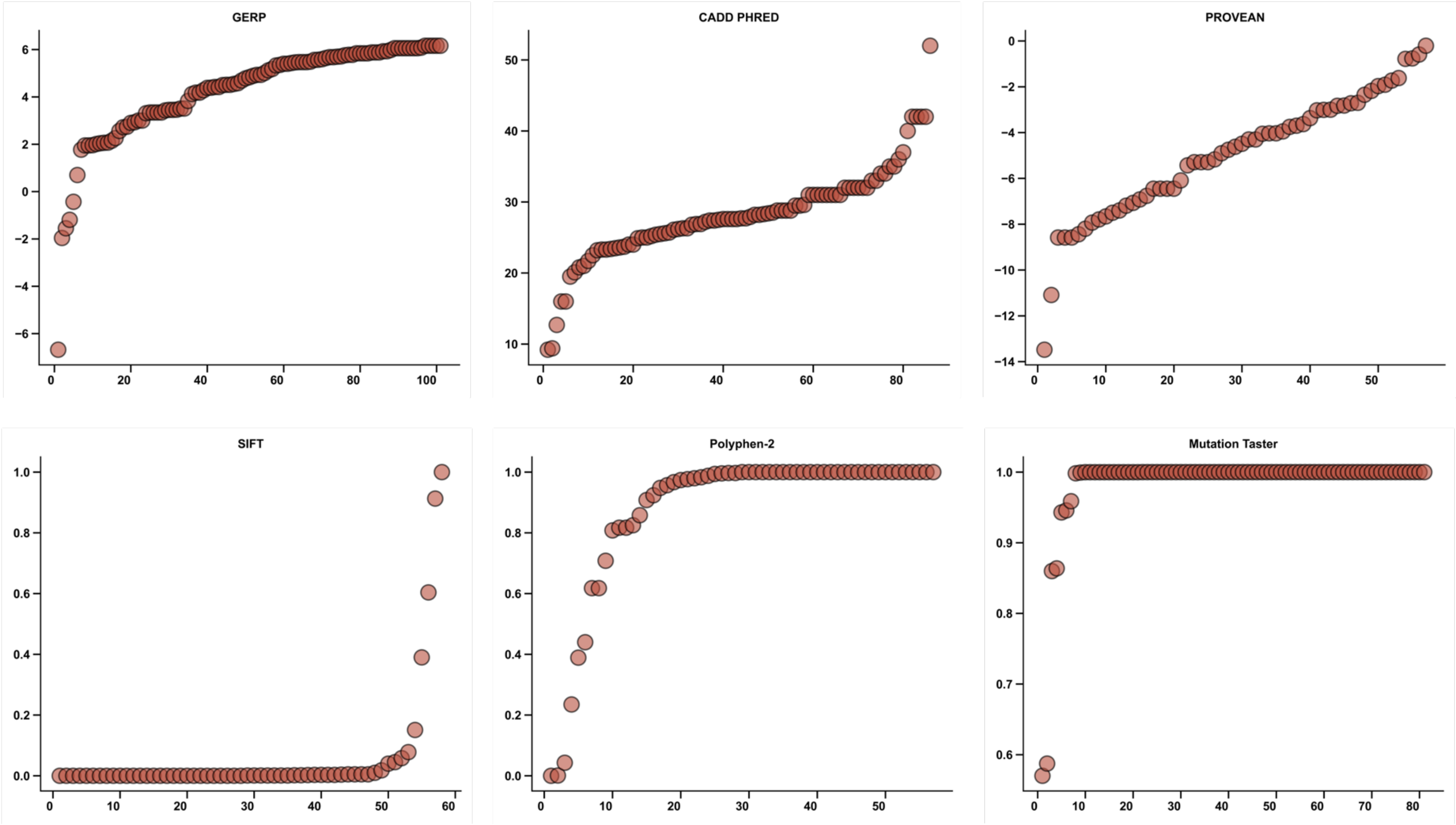

Supplementary Figure 7
Lollipop plots of genes containing novel variants

Throughout, the following key applies:

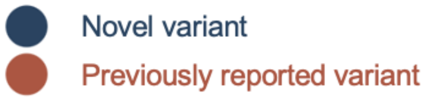

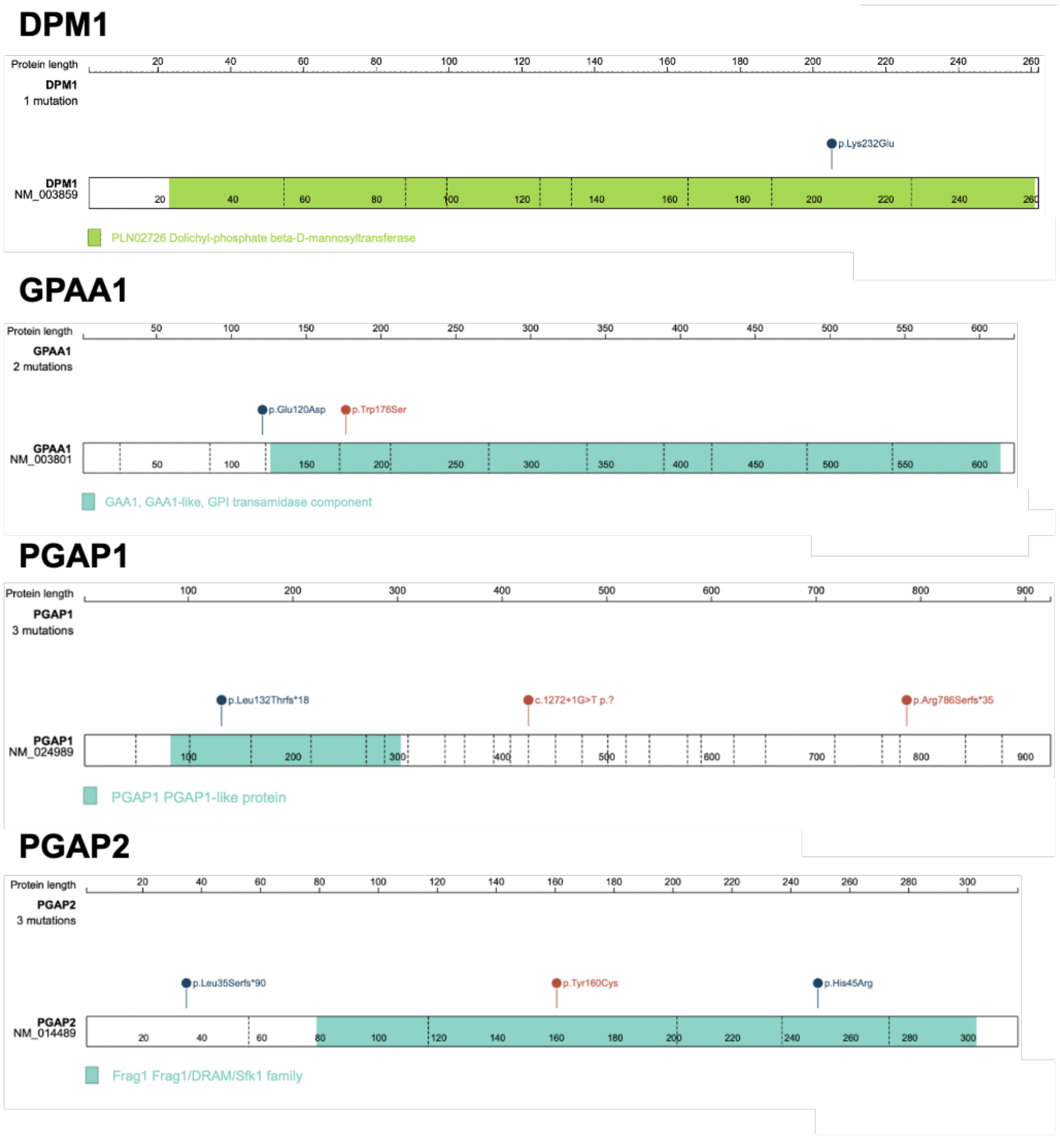

PIGA

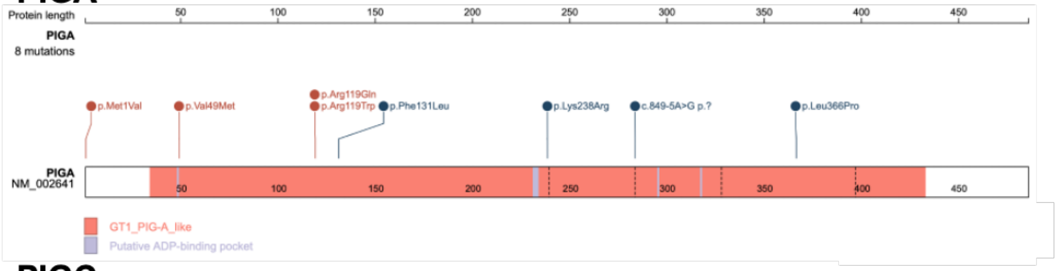

PIGC

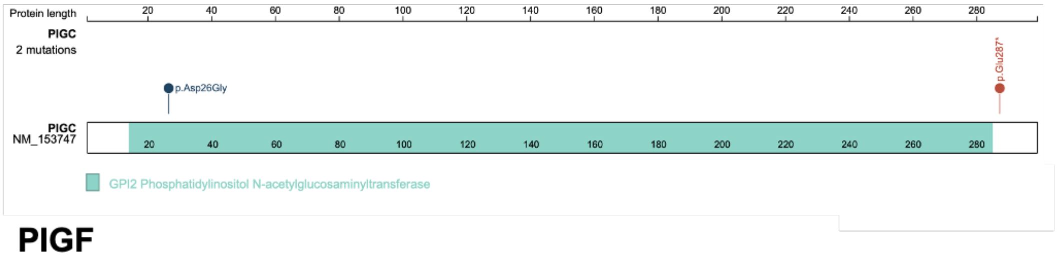

PIGF

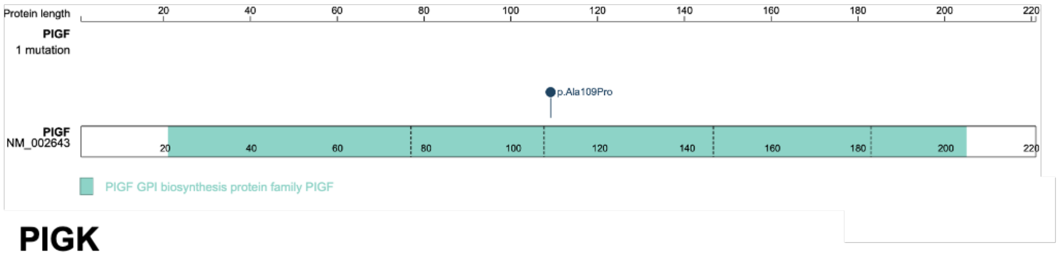

PIGK

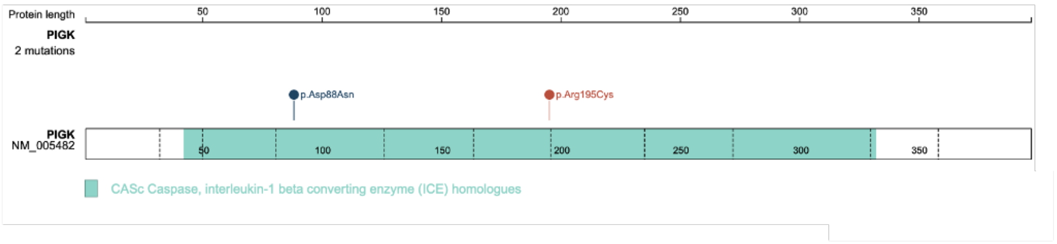

PIGL

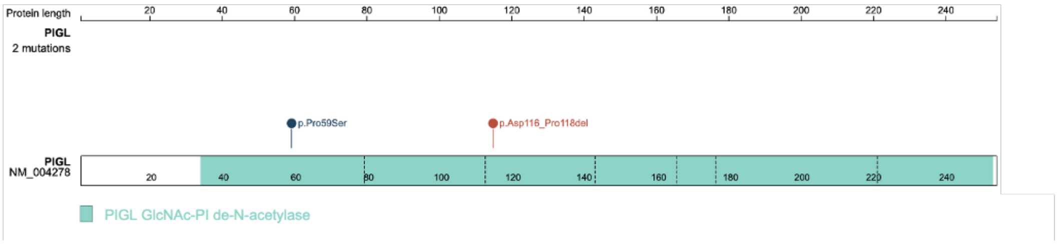

PIGN

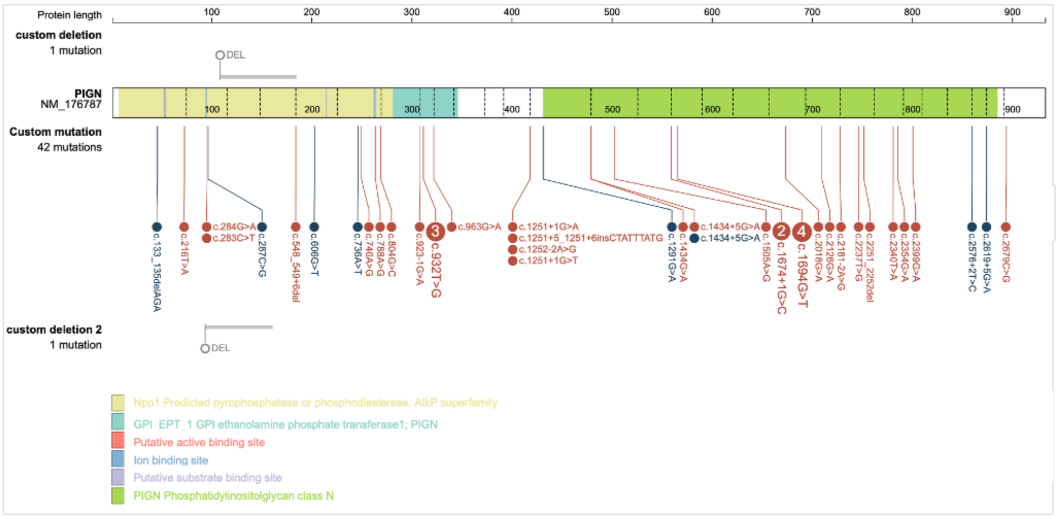
Protein length
100
200
300
400
500
600
700
800
900
custom deletion
1 mutation
DEL
PIGN
NM_176787
Custom mutation
42 mutations
c.133_135delAGA
c.216T>A
c.284G>A
c.283C>T
c.287C>G
c.548_549+6del
c.606G>T
c.736A>T
c.746A>G
c.788A>G
c.804G>C
c.923-1G>A
3
c.932T>G
c.963G>A
c.1251+1G>A
c.1251+5_1251+6insCTATTTATG
c.1252-2A>G
c.1251+1G>T
c.1291G>A
c.1434G>A
c.1434+5G>A
c.1434+5G>A
c.1505A>G
2
c.1674+1G>C
4
c.1694G>T
c.2018G>A
c.2126G>A
c.2181-2A>G
c.2237T>G
c.2251_2252del
c.2340T>A
c.2354G>A
c.2399G>A
c.2576+2T>C
c.2619+5G>A
c.2679C>G
custom deletion 2
1 mutation
DEL
Npp1 Predicted pyrophosphatase or phosphodiesterase, AlkP superfamily
GPI_EPT_1 GPI ethanolamine phosphate transferase1; PIGN
Putative active binding site
Ion binding site
Putative substrate binding site
PIGN Phosphatidylinositolglycan class N

PIGV

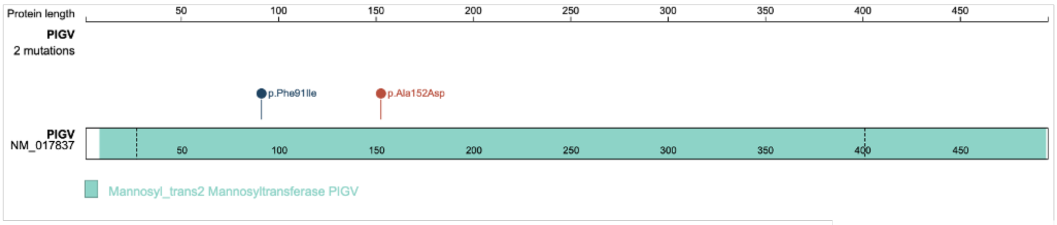
Protein length
50
100
150
200
250
300
350
400
450
PIGV
2 mutations
p.Phe91Ile
p.Ala152Asp
PIGV
NM_017837
Mannosyl_trans2 Mannosyltransferase PIGV

Supplementary Figure 8
Prevalence of core phenotypic features in our cohort compared to the last systematic review of IGD phenotypic features (Bellai-Dussault *et al*. *Clin Genet*. 2018)

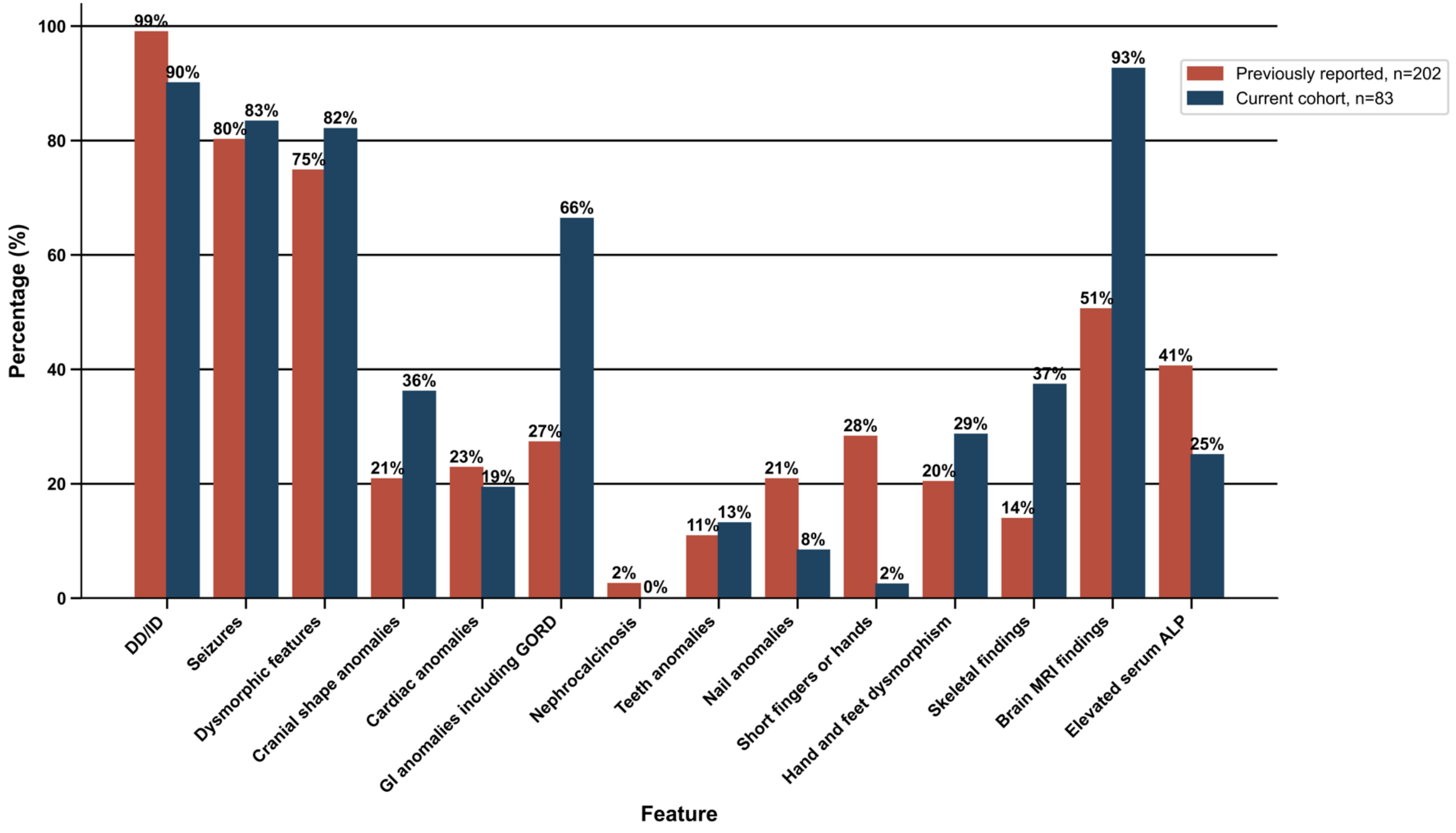

PIGX-IGD: clinical delineation

A female of Asian descent (F-67) presented at 1 year and 3 months of age with generalised onset myoclonic seizures. EEGs showed periodic discharges, generalised epileptiform activity, and a burst-suppression pattern. Neurological examination identified strabismus, nystagmus, and hearing loss. The obstetric history was unremarkable and there were no discernible dysmorphic features. There were no motor symptoms or non-neurological involvement. Whole exome sequencing identified a single biallelic homozygous missense variant in *PIGX* (c.4G>T p.Ala2Ser), with a very low allele count across multiple variant frequency databases. ACMG-AMP classification, however, rendered the variant of unknown significance (PM2, PM3, PP4, BP4). At last clinical follow-up, the child had persistent seizures, was non-ambulant, and had severe ID. She was vocalising but formed no specific words. Given that the clinical phenotype was strongly suggestive of an IGD (with infantile-onset generalised seizures and severe developmental delay), that this was the only variant detected on whole exome sequencing, and that there is no data excluding pathogenicity, this variant was deemed clinically diagnostic upon consensus review. Thereby, *PIGX* is established as a potential candidate gene for human neurogenetic disease, though we note the requirement of independent verification and further interrogation, ideally with functional studies.

Phenotypic analysis and natural history of recurrent variants

***PIGG* c.1515G>A**

This missense mutation was compound heterozygous in two unrelated individuals (F-3 and F-46) and homozygous in one individual (F-51). All individuals exhibited a strikingly similar clinical phenotype: early-onset infantile seizures with rapid seizure control, normal MRI brains, and no dysmorphic features or multisystem involvement. Interestingly, the homozygous individual exhibited no hypotonia or weakness and developed normally – with no DD/ID or speech delay – whilst the compound heterozygous individuals exhibited hypotonia, weakness, mild-moderate intellectual disability, and speech delay. All individuals were ambulant at last clinical follow up.

***PIGN* c.1694G>T**

This missense mutation was compound heterozygous in four unrelated individuals (F-19, F-30, F-36, and F-63) of North American descent and resulted in a mild phenotype: early-onset childhood seizures with rapid seizure control in 2/4 individuals, normal MRI brains, and no significant dysmorphic features or multisystem involvement. All individuals were ambulant but had delayed speech at last clinical follow-up.

***PIGN* c.932T>G**

This missense mutation was compound heterozygous in three unrelated individuals (F-19, F-47, and F-48), two of European and one of North American descent, and also resulted in a mild phenotype: early-onset infantile seizures with rapid seizure control in 2/3 individuals, normal MRI brains, and no significant dysmorphic features or multisystem involvement. Interestingly, 2/3 individuals had dystonic movements. All individuals were ambulant but had delayed speech at last clinical follow-up.

Familial variants

Though not strictly recurrent, the compound heterozygous *PIGN* variants c.1434G>A and c.2354G>A in two brothers of European descent (F-50) resulted in a particularly severe clinical phenotype, with early onset neonatal epilepsy, IESS, and intractable status epilepticus leading to death in the second year of life. Both individuals also had hypotonia, tremors, dyskinetic movement, and cortical visual impairment with grossly abnormal brain MRI exhibiting all core features (Figure 4A). Non-neurological findings in both individuals included patent foramen ovale, hydronephrosis, bitemporal narrowing, short arthrogrypotic hands and feet, and hypoplastic nails affecting the feet more than the hands (Figure 3A). Two novel variants were also seen in multiple siblings. The homozygous *PIGF* variant c.325G>C recurred in two brothers (F-61) of African descent, in whom the neurological phenotype was limited to ataxia of gait, nystagmus, and spasticity with cortical visual impairment. At last clinical follow-up, there were no seizures, mild DD/ID, ambulation with an aid, and staccato speech with no evident delay. Both exhibited bitemporal narrowing and malar flattening. The homozygous *PIGL* variant c.175C>T was also present in two siblings (F-68) of African descent. Both exhibited similar dysmorphic features: sparse eyebrows, depressed glabellae, exaggerated cupid's bow, and wide-spaced hypoplastic teeth, and neurological disease characterised by generalised early-onset infantile seizures with hypotonia, weakness, and ataxia. Seizure control was achieved in one individual. At last clinical follow-up, one individual was non-ambulant whilst the other was walking with an aid. Both exhibited delayed speech and severe DD/ID. Neither had non-neurological disease.

Flow cytometric experiments

Individuals with previously reported flow cytometric confirmation of variant pathogenicity

F-2, F-4, and F-5, *PIGT* c.1079G>T p.Gly360Val, previously reported in: Skauli N, Wallace S, Chiang SC, *et al*. Novel PIGT Variant in Two Brothers: Expansion of the Multiple Congenital Anomalies-Hypotonia Seizures Syndrome 3 Phenotype. *Genes (Basel)*. 2016;7(12):108.

F-7, *PIGK* c.262G>A p.Asp88Asn, previously reported in: Nguyen TTM, Murakami Y, Mobilio S, et al. Bi-allelic Variants in the GPI Transamidase Subunit PIGK Cause a Neurodevelopmental Syndrome with Hypotonia, Cerebellar Atrophy, and Epilepsy. *Am J Hum Genet*. 2020;106(4):484-495.

F-13, *PIGB* c.847-10A>G p.(?), previously reported in: Murakami Y, Nguyen TTM, Baratang N, *et al*. Mutations in PIGB Cause an Inherited GPI Biosynthesis Defect with an Axonal Neuropathy and Metabolic Abnormality in Severe Cases. *Am J Hum Genet*. 2019;105(2):384-394.

F-16, *PIGA* c.355C>T p.Arg119Trp, previously reported in: Kato M, Saitsu H, Murakami Y, *et al*. PIGA mutations cause early-onset epileptic encephalopathies and distinctive features. *Neurology*. 2014;82(18):1587-1596.

F-22, *PGAP3* c.914A>G p.Asp305Gly, previously reported in: Howard MF, Murakami Y, Pagnamenta AT, *et al*. Mutations in PGAP3 impair GPI-anchor maturation, causing a subtype of hyperphosphatasia with mental retardation. *Am J Hum Genet*. 2014;94(2):278-287.

F-40 and F-41, *PIGP* c.384delA p.Glu129Asnfs*34, previously reported in: Johnstone DL, Nguyen TT, Murakami Y, *et al*. Compound heterozygous mutations in the gene PIGP are associated with early infantile epileptic encephalopathy. *Hum Mol Genet*. 2017;26(9):1706-1715.

F-57, *PIGQ* c.1611del p.Arg538Alafs*24, previously reported in: Johnstone DL, Nguyen TTM, Zambonin J, *et al*. Early infantile epileptic encephalopathy due to biallelic pathogenic variants in PIGQ: Report of seven new subjects and review of the literature. *J Inherit Metab Dis*. 2020;43(6):1321-1332.

Individuals with newly reported flow cytometric confirmation of variant pathogenicity

In all patients, the reduction of GPI marker cell surface expression is characteristic of GPI anchor biosynthesis defects, thereby, confirming these defects in patient-derived cell lines (Supplementary Figure 9).

More specifically, for F-8, the expression of FLAER and GPI anchored proteins, such as CD16b and CD55, was reduced to 52%, 17%, and 43%, respectively, on granulocytes compared to the median of parents and the healthy control. Expression of FLAER, CD55, and CD16 was also reduced in the mother, compared to the father and the healthy unrelated control. Notably, CD16b expression was normal on granulocytes of the healthy mother. CD16b expression was not detected (probably due to a common polymorphism in CD16b) in the healthy control, it was, therefore, excluded from the analysis. F-50 also exhibited reduced expression with a bimodal distribution of the GPI-coupled proteins CD16 and CD24 on granulocytes and reduced expression of CD59 on erythrocytes when comparing the patient sample to controls. Similarly, the expression of the GPI anchored protein, CD59, was found to be reduced by 76%, 65%, 74% and 44% in patient fibroblast cell lines F-23, F-24, F-29, and F-34, respectively, when the means of patient and the healthy control were compared. Finally, the expression of the GPI-anchored proteins CD59 and DAF in *PIGL*-knockout HEK293 cells was rescued more efficiently, and to levels similar to those of the wildtype, when transfected with wildtype or mutant PIGL expression plasmids driven by a strong promoter (pME), but now with a weak promoter (pTA).

F-8

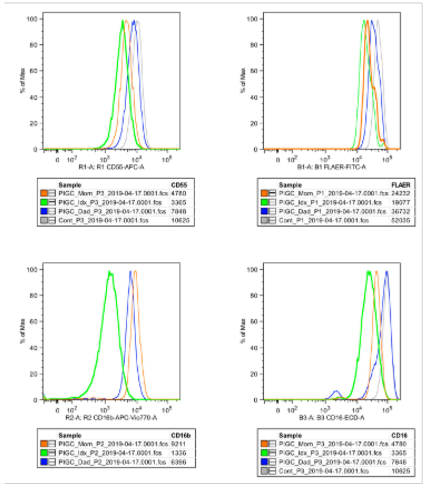

F-23

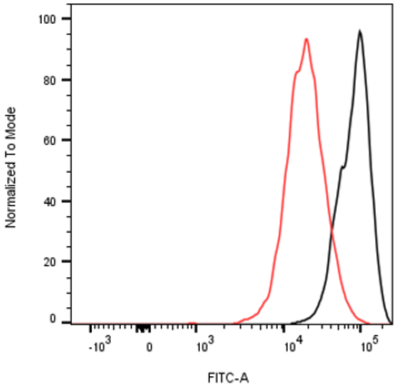

	Sample	CD59
■	Control	88528
■	CDG 110-1	21263

F-24

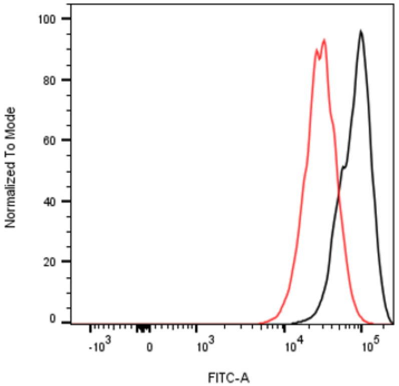

	Sample	CD59
■	Control	88528
■	CDG 137-1	30948

F-14

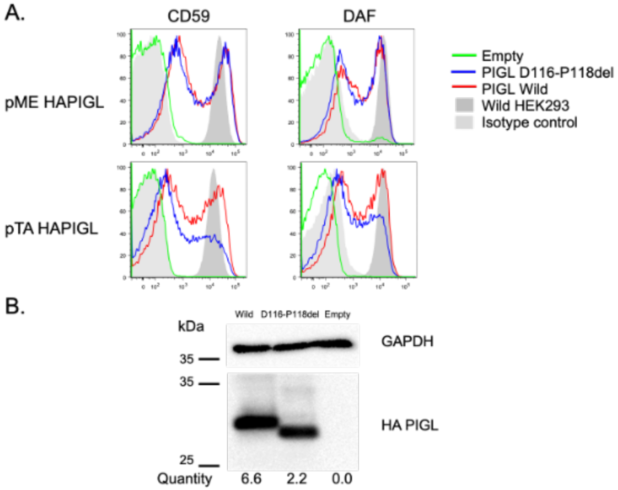

F-50

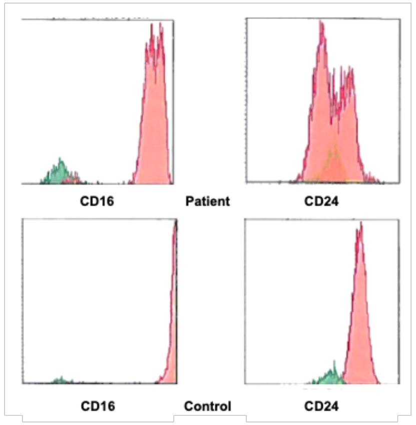

F-29

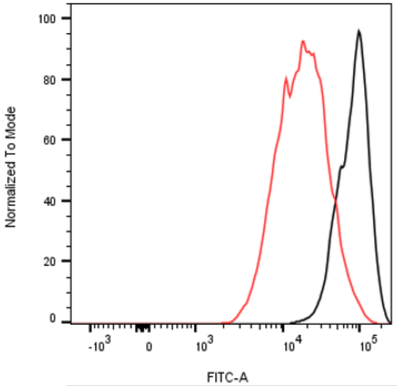

	Sample	CD59
■	Control	88528
■	CDG 147-1	22156

F-34

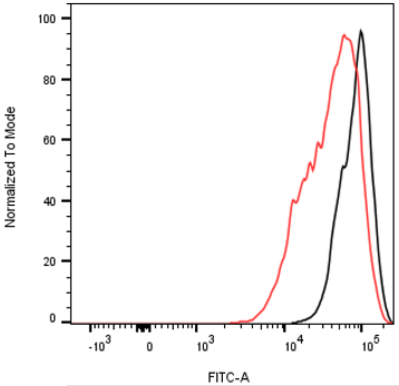

	Sample	CD59
■	Control	88528
■	CDG 161-1	49200

Supplementary Figure 9 | Measurement of GPI-anchored protein expression levels by flow cytometry in patient- derived granulocytes (F-8, F-50), patient-derived fibroblasts (F-23, F-24, F-29, F-34), and *PIGL*-knockout HEK293 cells (F-14). F-8: Histograms depict reduced expression of CD55, FLAER, and CD16b, all GPI anchored proteins, in patient granulocytes with *PIGC* gene defects as compared to the parents and healthy control. F-23, F-24, F-29, F-34: Histograms depict reduced expression of CD59, a GPI anchored protein, in patient-derived fibroblast cell lines with *PIGN* gene defects as compared to the control. F-50: Histograms depict reduced expression of CD16 (left) and CD24 (right), both GPI anchored proteins, in granulocytes of the patient (top) and the control (bottom). F-14: (A) Histograms depict the rescued expression of CD59 and DAF, both GPI-anchored proteins, on PIGL-knockout HEK293 cells transfected with wildtype or mutant PIGL expression plasmids driven by a strong promoter (pME) or a weak promoter (pTA). Mutant PIGL rescued GPI-AP expression at a similar level to the wildtype when driven by the strong promoter, though this was less efficient when driven by the weak promoter. (B) Cells transfected with pME HAPIGL in (A) were lysed and western blotting performed. After normalisation to luciferase activity and GAPDH, expression of the mutant protein was less than that of the wild-type protein.
